# Supplementary material for: Evidence-based modeling of combination control on Kenyan youth HIV/AIDS dynamics
Source: PLoS One. 2020 Nov 17;15(11):e0242491. doi: 10.1371/journal.pone.0242491 (PMC7671564; doi:10.1371/journal.pone.0242491)
Supplement: S1 Appendix — (PDF) [file pone.0242491.s001.pdf]

S1 Appendix. Endemic Equilibrium Expressions

Expressions for $g_{00}, g_{01}, ..., g_{11}$, $q_{01}, q_{02}, ..., q_{20}$, $h_{01}, h_{02}, ..., h_{20}$, $C_1, C_2, ..., C_5$ and $C_{11}, C_{21}, ..., C_{51}$ in section 2.3.3.

$$
\begin{cases}
g_{00} = \dfrac{\rho_{ct}^{f1}\,\mu_{f5} + \rho_{ct}^{f1}\,\rho_{ct}^{f}}{\mu_{f5}\mu_{f6} - \rho_{ct}^{f1}\,\rho_{ct}^{f}},\;\; g_{01} = \dfrac{\rho_{ct}^{f}(1+g_{00})}{\mu_{f5}},\;\; g_{02} = 1+\rho_{ht}^{m},\;\; g_{03} = \dfrac{\Lambda_{fa}\,\rho_{ht}^{m1}}{\mu_{f4}},\;\; g_{04} = \dfrac{\Lambda_{fu}\,\rho_{ht}^{m1}\rho_{ht}^{f}}{\mu_{f4}},\\
g_{05} = \dfrac{\Lambda_{fu}\,\rho_{ht}^{m}}{\mu_{f4}} + \dfrac{\Lambda_{fu}\,\rho_{ht}^{f}}{\mu_{f3}\,\mu_{f4}},\\
g_{06} = \dfrac{\rho_{ct}^{m1}\,\mu_{m5} + \rho_{ct}^{m1}\,\rho_{ct}^{m}}{\mu_{m5}\mu_{m6} - \rho_{ct}^{m1}\,\rho_{ct}^{m}},\;\; g_{07} = \dfrac{\rho_{ct}^{m}(1+g_{06})}{\mu_{m5}},\;\; g_{08} = 1+\rho_{ht}^{f},\;\; g_{09} = \dfrac{\Lambda_{ma}\,\rho_{ht}^{f1}}{\mu_{m4}},\;\; g_{10} = \dfrac{\Lambda_{mu}\,\rho_{ht}^{f1}\rho_{ht}^{m}}{\mu_{m4}},\\
g_{11} = \dfrac{\Lambda_{mu}\,\rho_{ht}^{f}}{\mu_{m4}} + \dfrac{\Lambda_{mu}\,\rho_{ht}^{m}}{\mu_{m3}\,\mu_{m4}},\\
q_{01} = N_f^*(\bar{\mu}_f + \delta_f) - (\Lambda_{fu} + \Lambda_{fa}),\;\; q_{02} = q_{01}\,g_{02}\,\rho_{ht}^{f},\\
q_{03} = (g_{02}\,q_{01}\,\mu_{f2} + q_{01}\,\mu_{f1}\rho_{ht}^{f}) - (\Lambda_{fu}\,\rho_{ht}^{f1}\,\delta_f + \Lambda_{fa}\,g_{02}\,\delta_f),\\
q_{04} = q_{01}\,\mu_{f1}\,\mu_{f2} - (\Lambda_{fu}\,\mu_{f2}\,\delta_f + \Lambda_{fa}\,\mu_{f1}\,\delta_f + \rho_{ht}^{f}\,\Lambda_{fu}\,\delta_f),\;\; q_{05} = \delta_f\,\rho_{ht}^{f}\,g_{00}\,g_{02},\\
q_{06} = g_{00}\,g_{02}\,\delta_f\,\mu_{f2} + g_{00}\,\delta_f\,\mu_{f1}\,\rho_{ht}^{f},\;\; q_{07} = g_{00}\,\delta_f\,\mu_{f1}\,\mu_{f2},\\
q_{08} = g_{02}\,g_{03}\,\rho_{ht}^{m1} + g_{05}\,\rho_{ht}^{m}\,\rho_{ht}^{m1},\\
q_{09} = g_{02}\,g_{03}\,\mu_{f2} + g_{03}\,\mu_{f1}\,\rho_{ht}^{m1} + g_{05}\,\mu_{f1}\,\rho_{ht}^{m1} + g_{05}\,\mu_{f2}\,\rho_{ht}^{m} + g_{04}\,\rho_{ht}^{m},\\
q_{10} = g_{03}\,\mu_{f1}\,\mu_{f2} + g_{05}\,\mu_{f1}\,\mu_{f2} + g_{04}\,\mu_{f2},\;\; q_{11} = g_{02}\,\rho_{ht}^{m}\,\rho_{ht}^{m1},\\
q_{12} = g_{02}\,\mu_{f2}\,\rho_{ht}^{m} + g_{02}\,\mu_{f2}\,\rho_{ht}^{m1} + \mu_{f1}\,\rho_{ht}^{m}\,\rho_{ht}^{m1},\\
q_{13} = g_{02}\,\mu_{f2}^2 + \mu_{f1}\,\mu_{f2}\rho_{ht}^{m} + \mu_{f1}\,\mu_{f2}\rho_{ht}^{m1},\;\; q_{14} = \mu_{f1}\,\mu_{f2}^2,\\
q_{15} = q_{08}\,q_{05} - q_{02}\,q_{11},\;\; q_{16} = q_{06}\,q_{08} + q_{05}\,q_{09} - (q_{02}\,q_{12} + q_{03}\,q_{11}),\\
q_{17} = q_{05}\,q_{10} + q_{06}\,q_{09} + q_{07}\,q_{08} - (q_{02}\,q_{13} + q_{03}\,q_{12} + q_{04}\,q_{11}),\\
q_{18} = q_{06}\,q_{10} + q_{07}\,q_{09} - (q_{02}\,q_{14} + q_{03}\,q_{13} + q_{04}\,q_{12}),\\
q_{19} = q_{07}\,q_{10} - (q_{03}\,q_{14} + q_{04}\,q_{13}),\;\; q_{20} = q_{04}\,q_{14},\\
h_{01} = N_m^*(\bar{\mu}_m + \delta_m) - (\Lambda_{mu} + \Lambda_{ma}),\;\; h_{02} = h_{01}\,g_{08}\,\rho_{ht}^{m},\\
h_{03} = (g_{08}\,h_{01}\,\mu_{m2} + h_{01}\,\mu_{m1}\rho_{ht}^{m}) - (\Lambda_{mu}\,\rho_{ht}^{m1}\,\delta_m + \Lambda_{ma}\,g_{08}\,\delta_m),\\
h_{04} = h_{01}\,\mu_{m1}\,\mu_{m2} - (\Lambda_{mu}\,\mu_{m2}\,\delta_m + \Lambda_{ma}\,\mu_{m1}\,\delta_m + \rho_{ht}^{m}\,\Lambda_{mu}\,\delta_m),\;\; h_{05} = \delta_m\,\rho_{ht}^{m}\,g_{06}\,g_{08},\\
h_{06} = g_{06}\,g_{08}\,\delta_m\,\mu_{m2} + g_{06}\,\delta_m\,\mu_{m1}\,\rho_{ht}^{m},\;\; h_{07} = g_{06}\,\delta_m\,\mu_{m1}\,\mu_{m2},\\
h_{08} = g_{08}\,g_{09}\,\rho_{ht}^{f1} + g_{11}\,\rho_{ht}^{f}\,\rho_{ht}^{f1},\\
h_{09} = g_{08}\,g_{09}\,\mu_{m2} + g_{09}\,\mu_{m1}\,\rho_{ht}^{f1} + g_{11}\,\mu_{m1}\,\rho_{ht}^{f1} + g_{11}\,\mu_{m2}\,\rho_{ht}^{f} + g_{10}\,\rho_{ht}^{f},\\
h_{10} = g_{09}\,\mu_{m1}\,\mu_{m2} + g_{11}\,\mu_{m1}\,\mu_{m2} + g_{10}\,\mu_{m2},\;\; h_{11} = g_{08}\,\rho_{ht}^{f}\,\rho_{ht}^{f1},\\
h_{12} = g_{08}\,\mu_{m2}\,\rho_{ht}^{f} + g_{08}\,\mu_{m2}\,\rho_{ht}^{f1} + \mu_{m1}\,\rho_{ht}^{f}\,\rho_{ht}^{f1},\\
h_{13} = g_{08}\,\mu_{m2}^2 + \mu_{m1}\,\mu_{m2}\rho_{ht}^{f} + \mu_{m1}\,\mu_{m2}\rho_{ht}^{f1},\;\; h_{14} = \mu_{m1}\,\mu_{m2}^2,\\
h_{15} = h_{08}\,h_{05} - h_{02}\,h_{11},\;\; h_{16} = h_{06}\,h_{08} + h_{05}\,h_{09} - (h_{02}\,h_{12} + h_{03}\,h_{11}),\\
h_{17} = h_{05}\,h_{10} + h_{06}\,h_{09} + h_{07}\,h_{08} - (h_{02}\,h_{13} + h_{03}\,h_{12} + h_{04}\,h_{11}),\\
h_{18} = h_{06}\,h_{10} + h_{07}\,h_{09} - (h_{02}\,h_{14} + h_{03}\,h_{13} + h_{04}\,h_{12}),\\
h_{19} = h_{07}\,h_{10} - (h_{03}\,h_{14} + h_{04}\,h_{13}),\;\; h_{20} = h_{04}\,h_{14},\\
C_1 = \dfrac{q_{16}}{q_{15}},\;\; C_2 = \dfrac{q_{17}}{q_{15}},\;\; C_3 = \dfrac{q_{18}}{q_{15}},\;\; C_4 = \dfrac{q_{19}}{q_{15}},\;\; C_5 = \dfrac{q_{20}}{q_{15}},\\
\\
C_{11} = \dfrac{h_{16}}{h_{15}},\;\; C_{21} = \dfrac{h_{17}}{h_{15}},\;\; C_{31} = \dfrac{h_{18}}{h_{15}},\;\; C_{41} = \dfrac{h_{19}}{h_{15}},\;\; C_{51} = \dfrac{h_{20}}{h_{15}}.
\end{cases}
\tag{15}
$$
