# Supplementary material for: Evidence-based modeling of combination control on Kenyan youth HIV/AIDS dynamics
Source: PLoS One. 2020 Nov 17;15(11):e0242491. doi: 10.1371/journal.pone.0242491 (PMC7671564; doi:10.1371/journal.pone.0242491)
Supplement: S2 Appendix — (PDF) [file pone.0242491.s002.pdf]

S2 Appendix. Single-Sex Model Description and Parameter Values

Equation 16 gives the single-sex model incidence rates and exit rates presented in equation 14.

$$\begin{cases} \beta_u = \dfrac{c\,\gamma}{N_y}\left[I_u + \alpha_c\rho_c I_a + (\alpha_c\rho_c + \alpha_t\rho_t)T_u\right], \\ \beta_a = \dfrac{c\,\gamma}{N_y}\left[I_u + \alpha_c\rho_c I_a + (\alpha_c\rho_c + \alpha_t\rho_t)T_u\right]\alpha_{ht}\,\rho_{ht}, \\ \tilde{\beta}_a = \dfrac{c\,\gamma}{N_y}\left[I_u + \alpha_c\rho_c I_a + (\alpha_c\rho_c + \alpha_t\rho_t)T_u\right]\alpha_{ht}^1\,\rho_{ht}, \\ \mu_1 = \rho_{ht} + \mu + \sigma,\ \mu_2 = \mu + \sigma,\ \mu_3 = \rho_{ht} + \mu + \sigma + \delta,\ \mu_4 = \rho_{ct} + \rho_{ct}^1 + \mu + \sigma + \delta, \\ \mu_5 = \rho_{ct}^1 + \bar{\mu} + \delta,\ \mu_6 = \rho_{ct} + \mu + \sigma. \end{cases} \tag{16}$$
