# Supplementary material for: Evidence-based modeling of combination control on Kenyan youth HIV/AIDS dynamics
Source: PLoS One. 2020 Nov 17;15(11):e0242491. doi: 10.1371/journal.pone.0242491 (PMC7671564; doi:10.1371/journal.pone.0242491)
Supplement: S1 Table — (PDF) [file pone.0242491.s003.pdf]

S1 Table. Description of Single-Sex Model State variables.

Variable	Description
S_u	Susceptible youth who have never tested for HIV/AIDS
S_a	Susceptible youth who have ever tested for HIV/AIDS
I_u	Infected youth who have never tested for HIV/AIDS
I_a	Infected youth who have ever tested for HIV/AIDS
T_u	Infected aware youth who are not adherent to ART or consistent condom use
T_a	Infected aware youth who are adherent to ART and use condoms consistently
