# Supplementary material for: Evidence-based modeling of combination control on Kenyan youth HIV/AIDS dynamics
Source: PLoS One. 2020 Nov 17;15(11):e0242491. doi: 10.1371/journal.pone.0242491 (PMC7671564; doi:10.1371/journal.pone.0242491)
Supplement: S2 Table — (PDF) [file pone.0242491.s004.pdf]

**S2 Table. Description of Single-Sex Model Parameters.**

| Parameter | Description |
|---|---|
| $\Lambda_u$ | Natural birth and maturity rate of susceptible youth unaware of their HIV status |
| $\Lambda_a$ | Natural birth and maturity rate of susceptible youth aware of their HIV status |
| $\rho_{ht}$ | Youth HIV testing rates |
| $\rho_t$ | Youth adherence rate to anti-retroviral therapy treatment |
| $\rho_c$ | Youth condom use rate |
| $\mu$ | Natural death rate of youth respectively |
| $\gamma$ | Probability of youth transmission risk |
| $\delta$ | Disease induced deaths in youth |
| $c$ | Youth sexual contact rate |
| $\alpha_{ht}$, $\alpha_{ht}^1$ | Factors negatively and positively influencing HIV testing rate among the youth |
| $\alpha_c$, $\alpha_c^1$ | Factors negatively and positively influencing condom use rate among the youth |
| $\alpha_t$, $\alpha_t^1$ | Factors negatively and positively influencing ART adherence rate among the youth |
| $\sigma$ | Exit rate of youth upon turning 24 years |
