# Supplementary material for: Evidence-based modeling of combination control on Kenyan youth HIV/AIDS dynamics
Source: PLoS One. 2020 Nov 17;15(11):e0242491. doi: 10.1371/journal.pone.0242491 (PMC7671564; doi:10.1371/journal.pone.0242491)
Supplement: S3 Table — (PDF) [file pone.0242491.s005.pdf]

S3 Table. Parameter Values for the Single-Sex Model, $\tilde{\gamma} = c\gamma$.

Parameter	Value	Unit	Source
Λ_u, Λ_a	60.476325, 100.55365	$year^{-1}$	Data Estimated
μ	0.0095859	$year^{-1}$	Data Estimated
$\tilde{\gamma}$	3.17245525	$year^{-1}$	Data Estimated
δ	0.0095	$year^{-1}$	Data Estimated
σ	0.041667	$year^{-1}$	Calculated
ρ_{ht}	0.48	$year^{-1}$	Data Estimated
ρ_c	0.3	$year^{-1}$	Data Estimated
ρ_t	0.1	$year^{-1}$	Data Estimated
α_{ht}, α_c, α_t	0.4, 0.27, 0.1	$year^{-1}$	Estimated
α_{ht}^1, α_c^1, α_t^1	0.78, 0.8, 0.75	$year^{-1}$	Estimated
