# Supplementary material for: Evidence-based modeling of combination control on Kenyan youth HIV/AIDS dynamics
Source: PLoS One. 2020 Nov 17;15(11):e0242491. doi: 10.1371/journal.pone.0242491 (PMC7671564; doi:10.1371/journal.pone.0242491)
Supplement: Attachment — Submitted filename: PONE-D-20-04014_reviewer.pdf [file pone.0242491.s007.pdf]

PLOS ONE

Evidence-based modeling of combinatory control on Kenyan youth HIV/AIDS dynamics

--Manuscript Draft--

**Manuscript Number:**	PONE-D-20-04014
**Article Type:**	Research Article
**Full Title:**	Evidence-based modeling of combinatory control on Kenyan youth HIV/AIDS dynamics
**Short Title:**	Modeling HIV/AIDS dynamics among the Kenyan Youth
**Corresponding Author:**	Marilyn Ronoh<br>University of Nairobi<br>Nairobi, Kenya KENYA
**Keywords:**	HIV/AIDS; AGYW; ABYM; HIV testing; condom use; antiretroviral therapy; combinatory control
**Abstract:**	We formulate a sex-structured deterministic model to study the effects of varying HIV/AIDS testing rates, condom use rates and ART adherence rates among Adolescent Girls and Young Women (AGYW) and, Adolescent Boys and Young Men (ABYM) populations in Kenya. Attitudes influencing the Kenyan youth HIV/AIDS controls both positively and negatively were considered. Using the 2012 Kenya AIDS Indicator Survey (KAIS) microdata we constructed our model, which we fitted to the UNAIDS-Kenya youth prevalence estimates to understand factors influencing AGYW/ABYM HIV/AIDS prevalence trends. While highly efficacious combinatory control approach significantly reduces HIV/AIDS prevalence rates among the AGYW/ABYM, the disease remains endemic provided infected unaware sexual interactions persist. Disproportional gender-wise attitudes towards HIV/AIDS controls play a key role in reducing the Kenyan youth HIV/AIDS prevalence trends.
**Order of Authors:**	Marilyn Ronoh
	Faraimunashe Chirove
	Josephine Wairimu
	Wandera Ogana
**Additional Information:**	
**Question**	**Response**
**Financial Disclosure**<br><br>Enter a financial disclosure statement that describes the sources of funding for the work included in this submission. Review the submission guidelines for detailed requirements. View published research articles from *PLOS ONE* for specific examples.<br><br>This statement is required for submission and **will appear in the published article** if the submission is accepted. Please make sure it is accurate.	The authors received no specific funding for this work

Powered by Editorial Manager® and ProduXion Manager® from Aries Systems Corporation

**Unfunded studies**<br>Enter: *The author(s) received no specific funding for this work.*<br><br>**Funded studies**<br>Enter a statement with the following details:<br>• Initials of the authors who received each award<br>• Grant numbers awarded to each author<br>• The full name of each funder<br>• URL of each funder website<br>• Did the sponsors or funders play any role in the study design, data collection and analysis, decision to publish, or preparation of the manuscript?<br>• **NO** - Include this sentence at the end of your statement: *The funders had no role in study design, data collection and analysis, decision to publish, or preparation of the manuscript.*<br>• **YES** - Specify the role(s) played.<br><br>* typeset	
**Competing Interests**<br><br>Use the instructions below to enter a competing interest statement for this submission. On behalf of all authors, disclose any competing interests that could be perceived to bias this work—acknowledging all financial support and any other relevant financial or non-financial competing interests.<br><br>This statement **will appear in the published article** if the submission is accepted. Please make sure it is accurate. View published research articles from *PLOS ONE* for specific examples.	The authors have declared that no competing interests exist

Powered by Editorial Manager® and ProduXion Manager® from Aries Systems Corporation

**NO authors have competing interests**<br><br>Enter: *The authors have declared that no competing interests exist.*<br><br>**Authors with competing interests**<br><br>Enter competing interest details beginning with this statement:<br><br>*I have read the journal's policy and the authors of this manuscript have the following competing interests: [insert competing interests here]*<br><br>* typeset	
**Ethics Statement**<br><br>Enter an ethics statement for this submission. This statement is required if the study involved:<br><br>• Human participants<br>• Human specimens or tissue<br>• Vertebrate animals or cephalopods<br>• Vertebrate embryos or tissues<br>• Field research<br><br>Write "N/A" if the submission does not require an ethics statement.<br><br>General guidance is provided below. Consult the submission guidelines for detailed instructions. **Make sure that all information entered here is included in the Methods section of the manuscript.**	N/A

Powered by Editorial Manager® and ProduXion Manager® from Aries Systems Corporation

**Format for specific study types**<br><br>**Human Subject Research (involving human participants and/or tissue)**<br>• Give the name of the institutional review board or ethics committee that approved the study<br>• Include the approval number and/or a statement indicating approval of this research<br>• Indicate the form of consent obtained (written/oral) or the reason that consent was not obtained (e.g. the data were analyzed anonymously)<br><br>**Animal Research (involving vertebrate animals, embryos or tissues)**<br>• Provide the name of the Institutional Animal Care and Use Committee (IACUC) or other relevant ethics board that reviewed the study protocol, and indicate whether they approved this research or granted a formal waiver of ethical approval<br>• Include an approval number if one was obtained<br>• If the study involved *non-human primates*, add *additional details* about animal welfare and steps taken to ameliorate suffering<br>• If anesthesia, euthanasia, or any kind of animal sacrifice is part of the study, include briefly which substances and/or methods were applied<br><br>**Field Research**<br><br>Include the following details if this study involves the collection of plant, animal, or other materials from a natural setting:<br>• Field permit number<br>• Name of the institution or relevant body that granted permission	
**Data Availability**<br><br>Authors are required to make all data underlying the findings described fully available, without restriction, and from the time of publication. PLOS allows rare exceptions to address legal and ethical concerns. See the PLOS Data Policy and FAQ for detailed information.	No - some restrictions will apply

Powered by Editorial Manager® and ProduXion Manager® from Aries Systems Corporation

A Data Availability Statement describing where the data can be found is required at submission. Your answers to this question constitute the Data Availability Statement and **will be published in the article**, if accepted.<br><br>**Important:** Stating 'data available on request from the author' is not sufficient. If your data are only available upon request, select 'No' for the first question and explain your exceptional situation in the text box.<br><br>Do the authors confirm that all data underlying the findings described in their manuscript are fully available without restriction?	
**Describe where the data may be found in full sentences. If you are copying our sample text, replace any instances of XXX with the appropriate details.**<br><br>• If the data are **held or will be held in a public repository**, include URLs, accession numbers or DOIs. If this information will only be available after acceptance, indicate this by ticking the box below. For example: *All XXX files are available from the XXX database (accession number(s) XXX, XXX.*).<br>• If the data are all contained **within the manuscript and/or Supporting Information files**, enter the following: *All relevant data are within the manuscript and its Supporting Information files.*<br>• If neither of these applies but you are able to provide **details of access elsewhere**, with or without limitations, please do so. For example:<br><br>*Data cannot be shared publicly because of [XXX]. Data are available from the XXX Institutional Data Access / Ethics Committee (contact via XXX) for researchers who meet the criteria for access to confidential data.*<br><br>*The data underlying the results presented in the study are available from (include the name of the third party*	Data cannot be shared publicly because of copyright issues. Data are available from the National Data Archive (KeNADA) in the Kenya National Bureau of Statistics website (http://54.213.151.253/nada/index.php/catalog/94/accesspolicy) for researchers who meet the criteria for access to confidential data.

and contact information or URL).<br>• This text is appropriate if the data are owned by a third party and authors do not have permission to share the data.<br><br>* typeset	
Additional data availability information:	

Powered by Editorial Manager® and ProduXion Manager® from Aries Systems Corporation

Manuscript

Click here to download Manuscript ronoh marilyn_plos manuscript.pdf

Evidence-based modeling of combinatory control on Kenyan youth HIV/AIDS dynamics

Marilyn Ronoh[1*◕], Faraimunashe Chirove[2,3◕], Josephine Wairimu[1◕], Wandera Ogana[1◕]

1 School of Mathematics, University of Nairobi, Nairobi, Kenya
2 School of Mathematics, Statistics and Computer Science, University of KwaZulu-Natal, Durban, South Africa
3 Department of Mathematics and Applied Mathematics, University of Johannesburg, Johannesburg, South Africa

◕These authors contributed equally to this work.
* mcronoh1@gmail.com

Abstract

We formulate a sex-structured deterministic model to study the effects of varying HIV/AIDS testing rates, condom use rates and ART adherence rates among Adolescent Girls and Young Women (AGYW) and, Adolescent Boys and Young Men (ABYM) populations in Kenya. Attitudes influencing the Kenyan youth HIV/AIDS controls both positively and negatively were considered. Using the 2012 Kenya AIDS Indicator Survey (KAIS) microdata we constructed our model, which we fitted to the UNAIDS-Kenya youth prevalence estimates to understand factors influencing AGYW/ABYM HIV/AIDS prevalence trends. While highly efficacious combinatory control approach significantly reduces HIV/AIDS prevalence rates among the AGYW/ABYM, the disease remains endemic provided infected unaware sexual interactions persist. Disproportional gender-wise attitudes towards HIV/AIDS controls play a key role in reducing the Kenyan youth HIV/AIDS prevalence trends.

1 Introduction

Kenya's HIV epidemic ranks fourth worldwide with its general population affected most alongside risk groups such as sex workers, people who inject drugs, men who have sex with men and recently, the youth population [1,2]. Two decades of successful combination control efforts such as HIV testing, public health education campaigns, condom usage, antiretroviral therapy (ART) among others has resulted in the country's significant reduction of the HIV/AIDS prevalence from 10.5% in 1996 to 5.9% in 2015 [3].

Integral to the ongoing fight against HIV/AIDS in Kenya is the component of HIV Counseling and Testing (HCT) with the Government of Kenya and International Development Partners substantially increasing voluntary counseling and testing (VCT) services in the country in the recent years [4]. Under the Adolescent Reproductive Health Development policy in the 2005-2015 Plan of Action the Government of Kenya sought to establish adolescent friendly voluntary counseling and testing services in a bid to improve and promote accessibility of youth friendly sexual and reproductive health services [5]. Scale up in innovative approaches to HIV/AIDS testing in the country include community based HIV/AIDS testing, door to door testing campaigns and most

recently, self-testing kits [6, 7]. Despite these great progress in increasing HIV/AIDS testing centers and new approaches to HIV/AIDS testing, combined effects of inadequate health services, poverty, sociodemographic characteristics, HIV testing behavior, difficult socio-cultural and psycho-social conditions heavily impact the adolescents and young adults volunteering to HIV testing [8–10]. There is significant gender disparity in factors associated with HIV/AIDS testing among the youth in Kenya with pregnant female youth required to test for HIV/AIDS due to advanced prevention of mother-to-child transmission(PMTCT) in the country compared to their male counterparts leading to female youth reporting higher HIV/AIDS testing rates in comparison to male youth of a similar age cohort [2, 3, 11].

Young people aged 15-24 in Kenya often engage in unprotected and unplanned sexual intercourse often resulting in sexually transmitted infections, pregnancies and HIV infections [3, 11–13]. While condom use offers dual protection against unplanned pregnancies and protection against HIV/AIDS infection, there is increasing decline in condom use among the youth in Kenya [11, 13]. Some of the factors influencing condom use among the Kenyan youth include perceived individual's risk, peer influence, partner betrayal and socio-cultural factors such as religion, communities, schools and families [3, 12–15]. Adolescents and young adults are easily influenced with their peers negative attitudes to condom use with male peers highly affected compared to female peers [16, 17]. Incorrect use of condoms in these population group places them at a higher risk of HIV/AIDS infection as many of them are experimenting with sex or under the influence of drugs or alcohol [12, 13]. While condom use among the adolescents and young adults remains inconsistent, condom use is generally higher among male youth compared to female youth due to the patriarchal society in Kenya where the male condom is the most preferred method with female youth reporting pressure from male partner not to use condoms [12, 13, 15]. External funding was responsible for most of the free condoms distribution in Kenya and recent cuts in donor funding has affected majority of the sexually active youth in Kenya who cannot afford to purchase condoms. [18].

Universal Test and Treat strategy by the World Health organization requires that all persons testing positive for HIV/AIDS be initiated on ART immediately irrespective of their CD4+ T cell count so as to achieve 90% diagnosis of all HIV positive persons with 90% of those positively diagnosed initiated on ART so as to achieve 90% viral load suppression [19]. Unfortunately, the adherence rates to ART is proving to be an uphill task among the adolescents and young adults in Kenya [20]. Factors influencing non adherence to ART among the youth in Kenya include stigma associated with disclosure of HIV/AIDS status, lack of adequate support from primary care givers and health workers, treatment fatigue, lack of adequate support structures in schools for youth living with HIV/AIDS, confidentiality breaches by health providers leading to disclosure of patients status to the community, fear of gossip and ridicule, financial constraints leading to failure to honor medical appointments or collect ART drugs and physical and emotional violence meted to orphaned perinatally infected youth by their care givers prompting them to fend for themselves or forcing them to street life [9, 10, 20, 21].

Models formulated for HIV/AIDS dynamics have so far informed strategic planning, implementation and evaluation of control programs [22–26]. Recent HIV/AIDS models have coupled interventions such as screening, anti-retroviral therapy (ART) treatment, Prep uptake and condom use [27–33]. Few of these models considered combination control strategies [34]. Real epidemiological data was used in [34–39] to predict HIV/AIDS prevalence subject to the considered controls.

We seek to show the effects of varying HIV/AIDS testing rates, condom use rates and antiretroviral adherence rates on the sex-structured AGYW/ABYM disease dynamics in Kenya subject to attitudes influencing disease control such as psycho-social conditions, sociodemographic and socio-cultural characteristics described earlier. Using the most recent UNAIDS-Kenya data we fit the AGYW/ABYM model prevalence under the three combinatory controls to their respective prevalence data for reliable prevalence predictions and model parameter estimation. HIV/AIDS prevalence among the Adolescent Girls and Young Women (AGYW) population aged 15-24 is high at 5.7% whereas the Adolescent Boys and Young Men (ABYM) population is low at 2.2% [2]. About 73.6% of adolescent girls and young women aged 15-24 tested for HIV/AIDS in 2015 [2]. Similarly, 56% of adolescent boys aged and young men aged 15-24 reported to have tested for HIV/AIDS that year [2]. Approximately 89% of the AGYW reported not using condoms in trusted sexual relations whereas 57.6% of ABYM used condoms at their first sexual encounter [2]. Out of the 268, 586 adolescents and young adults living with HIV/AIDS, 16% are yet to access anti-retroviral therapy (ART) [3]. This model formulation provides a low cost approach to identify key areas for intervention in the real world that could help in reducing new HIV/AIDS infections among the youth in Kenya.

2 Methods

2.1 Data Description

This section details the 2012 Kenya AIDS Indicator Survey description which was used to inform the model formulation described in section 2.2 and the UNAIDS-Kenya National Survey prevalence data description used for the model prevalence fit given in section 2.5.

2.1.1 Kenya AIDS Indicator Survey (KAIS) Data Description

We used the 2012 Kenya AIDS Indicator Survey (KAIS) micro-data obtained from the Kenya National Bureau of Statistics website [40] to construct our model as it included data on HIV testing, sexual behavior and HIV care and treatment of children and adults. Given our interest in HIV testing, sexual behavior and HIV care and treatment of adolescents and young adults, we concentrated only on the all adults and sexual partners data sets. The all adults data set comprised of adolescents and adults aged 15-64 years totaling to 10, 811 with 5,211 males and 5,600 females. The sex partner data set had information regarding sex partner's gender, sexual behavior and HIV/AIDS status. We considered the sex partner data set as we were interested in heterosexual partners. We combined the all adults data set with the sex partners data set and extracted adolescents and young adults aged 15-24 years. Thus, the combined data set comprised of 3,278 sexually active adolescents and young adults aged 15-24 years with 1,597 ABYM and 1,681 AGYW.

We generated a new variable for HIV/AIDS status knowledge from the combined data set based on HIV testing and it's structure included uninfected unaware, uninfected aware, infected unaware and infected aware. Uninfected aware population comprised of individuals who reported negative HIV/AIDS status and were KAIS confirmed negative and those who reported negative having tested for HIV/AIDS elsewhere. Uninfected unaware were individuals who reported never tested for HIV/AIDS and were KAIS confirmed negative and those who reported positive HIV/AIDS status and were KAIS confirmed negative. Infected aware included those AGYW / ABYM who reported positive HIV/AIDS status and were KAIS confirmed positive and those who self-reported positive having tested for HIV/AIDS elsewhere. We classified the infected unaware as those

who were HIV infected but reported negative and those who reported never tested for HIV/AIDS. Figures 1(a) and 1(b) gives the data summary for participant gender HIV status knowledge of the AGYW/ABYM. HIV/AIDS status knowledge is highest among AGYW in comparison to ABYM and this is consistent with literature findings described in section 1 (see figures 1(a) and 1(b)). Infected unaware AGYW/ABYM are way higher compared to infected aware AGYW/ ABYM which is worrying whereas uninfected aware AGYW/ ABYM are slightly higher than uninfected unaware AGYW/ ABYM (see figures 1(a) and 1(b)).

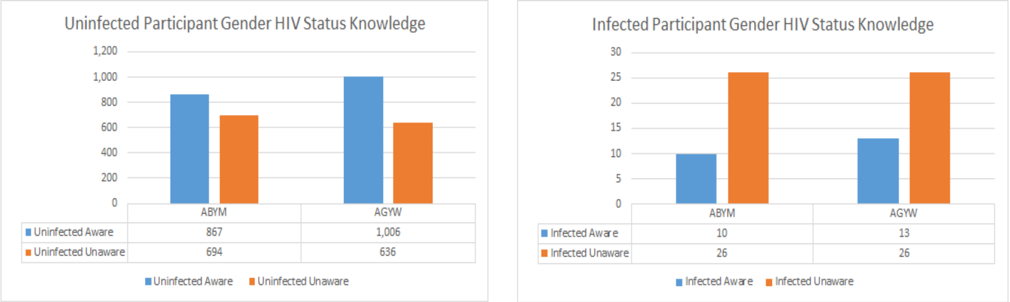

(a) Susceptible AGYW and ABYM HIV Status Knowledge

(b) Infected AGYW and ABYM HIV Status Knowledge

Fig 1. Participant Gender HIV Status Knowledge

The question around the use of condom every time with sexual partner was used to determine condom use patterns among the AGYW/ABYM and this was tabulated against their HIV status knowledge [40]. Figures 2(a) and 2(b) gives the data summary for participant gender condom use patterns with the AGYW/ABYM sexual partners.

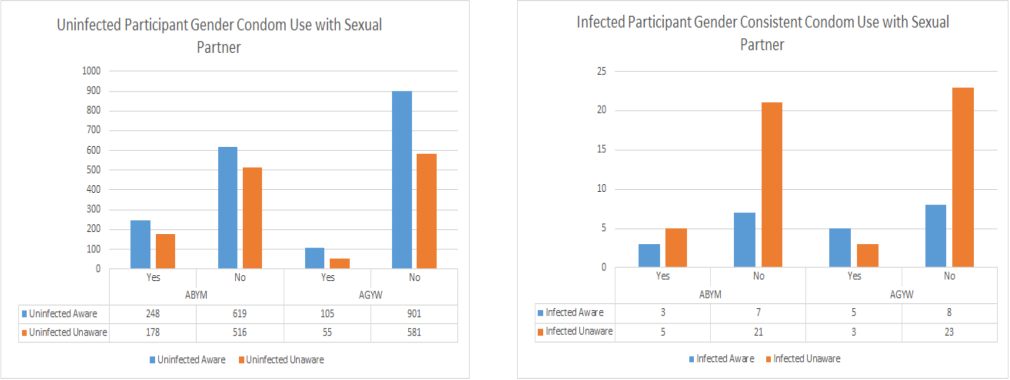

(a) Susceptible AGYW and ABYM Condom Use Patterns with Sexual Partner

(b) Infected AGYW and ABYM Condom Use Patterns with Sexual Partner

Fig 2. Participant Gender Condom Use Patterns with Sexual Partner

Consistent condom use patterns are way higher among the uninfected aware ABYM in comparison to uninfected aware AGYW (see figure 2(a)). However, most of the uninfected aware AGYW/ABYM fail to use condoms consistently with sexual partners with uninfected aware AGYW ranking highest (see figure 2(a)). While uninfected unaware populations fail to use condoms consistently with sexual partners too, they are slightly better in comparison to uninfected aware populations (see figure 2(a)). Infected aware AGYW use condoms more consistently with sexual partners when compared to

infected aware ABYM (see figure 2(b)). Infected unaware AGYW/ABYM inconsistent condom use with sexual partners are way higher than infected aware AGYW/ABYM populations (see figure 2(b)).

On ART adherence, the questions around currently using ART and daily ART usage were used to determine ART adherence among the infected AGYW/ABYM and this was also tabulated against their HIV status knowledge [40]. Figures 3(a) and 3(b) gives the data summary for participant gender HIV status knowledge and ART usage.

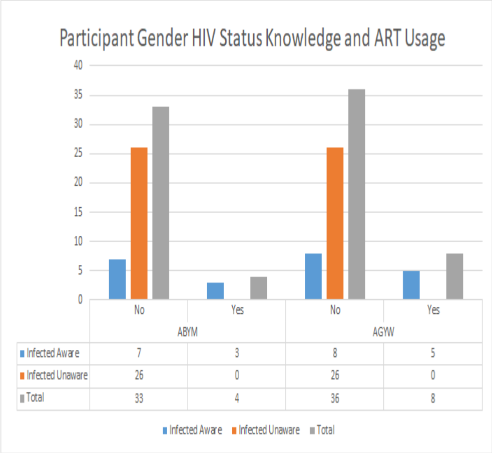

(a) Infected AGYW and ABYM on ART

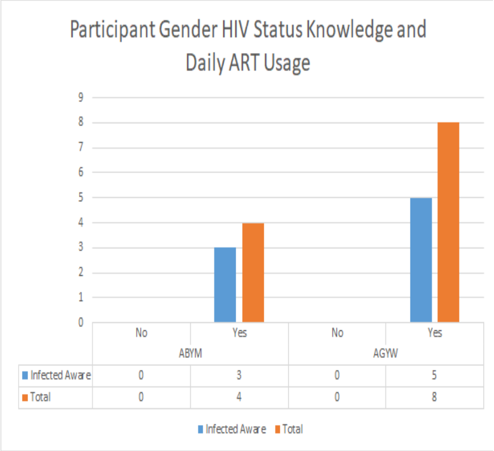

(b) Infected AGYW and ABYM Daily ART Usage

Fig 3. HIV/AIDS Infected Participant Gender ART Usage

Infected unaware populations are yet to be initiated on ART which is expected (see figures 3(a)) whereas few infected aware AGYW/ABYM are on ART (see figure 3(a)). Figure 3(b) shows AGYW/ABYM initiated on ART with daily use, which implies adherence to ART. However, majority of the infected aware AGYW/ABYM are yet to be initiated on ART (see figures 3(a)).

2.1.2 UNAIDS Data Description

The 2012 KAIS data informed the model formulation described in section 2.2. Given that the 2012 KAIS data was binary, it could only inform the model structure and some state variables initial conditions. Hence, we used the UNAIDS-Kenya National Survey quantitative data on Kenyan youth prevalence to fit the model prevalence for AGYW and ABYM populations. The model fit was also used to estimate the best parameter estimates for some of the model parameters and predict the AGYW and ABYM prevalence for the years 2019 - 2023. Tables 3 - 5 give the AGYW/ABYM UNAIDS-Kenya prevalence estimates and figures 4(a), 4(b) show the 1990 - 2018 UNAIDS-Kenya prevalence estimates for the Kenyan youth [41].

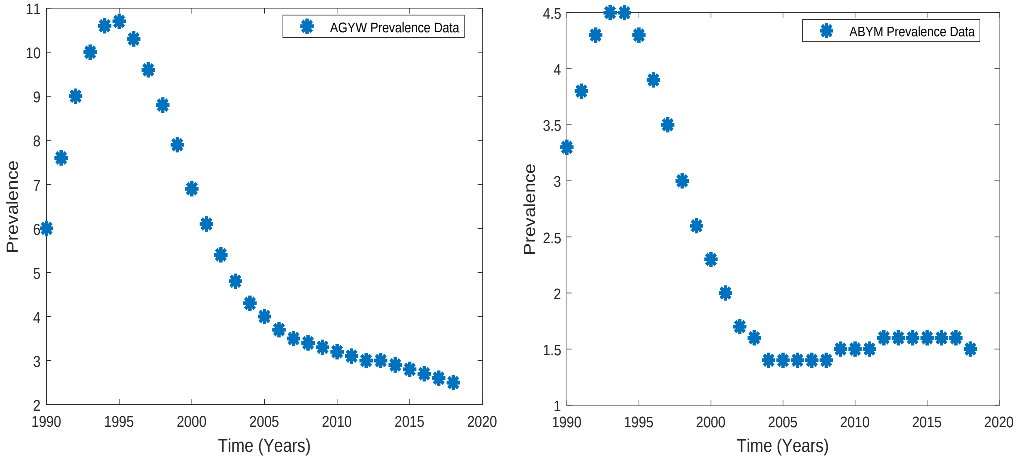

(a) AGYW UNAIDS-Kenya 1990 - 2018 Prevalence Estimates [41]

(b) ABYM UNAIDS-Kenya 1990 - 2018 Prevalence Estimates [41]

Fig 4. AGYW and ABYM UNAIDS-Kenya 1990-2018 Prevalence Estimates [41]

2.2 Model Formulation

We formulate a model describing HIV transmission dynamics in the AGYW and ABYM populations aged 15-24 with most of the state variables derived from the 2012 KAIS data described in section 2.1.1 [40]. While all the infected aware on ART treatment remained adherent in section 2.1.1 and figure 3, the model formulation considers the infected aware AGYW and ABYM populations on ART but are not adherent so as to make our model adaptable to non-adherence as the ART adherence rates among the infected aware youth in the KAIS data set was only for the 2012 data point. Section 1 highlights the need to model this population group as some of the infected aware youth on ART in general are not adherent to ART. Hence, we include this population group in the model formulation. We do not include the male population older than 24 years in this formulation as transactional sex in the 2012 KAIS population based survey was not common [42]. Hence, we primarily focus on the sexual behavior and use of HIV/AIDS controls among the sexually active youth aged 15-24 years.

The AGYW and ABYM populations are each categorized into six classes such that at time $t \geq 0$ there are susceptible AGYW, ABYM (S_{fu}, S_{mu}), infected AGYW, ABYM (I_{fu}, I_{mu}) who are not aware of their HIV status, susceptible AGYW, ABYM (S_{fa}, S_{ma}), infected AGYW, ABYM (I_{fa}, I_{ma}) who have tested for HIV/AIDS and are aware of their HIV status and use condoms consistently but are yet to be initiated on ART, infected AGYW, ABYM (T_{fu}, T_{mu}) who have tested for HIV/AIDS and are aware of their HIV status but use ART and condoms inconsistently and infected AGYW, ABYM (T_{fa}, T_{ma}) who have tested for HIV/AIDS and are aware of their HIV status and are adherent to ART and use condoms consistently. The total size of the AGYW and ABYM populations is given as $N_f = S_{fu} + S_{fa} + I_{fu} + I_{fa} + T_{fu} + T_{fa}$, $N_m = S_{mu} + S_{ma} + I_{mu} + I_{ma} + T_{mu} + T_{ma}$ respectively. $N = N_f + N_m$ is the total AGYW and ABYM population. Figure 5 represents the flow of individuals into different compartments in a single patch model.

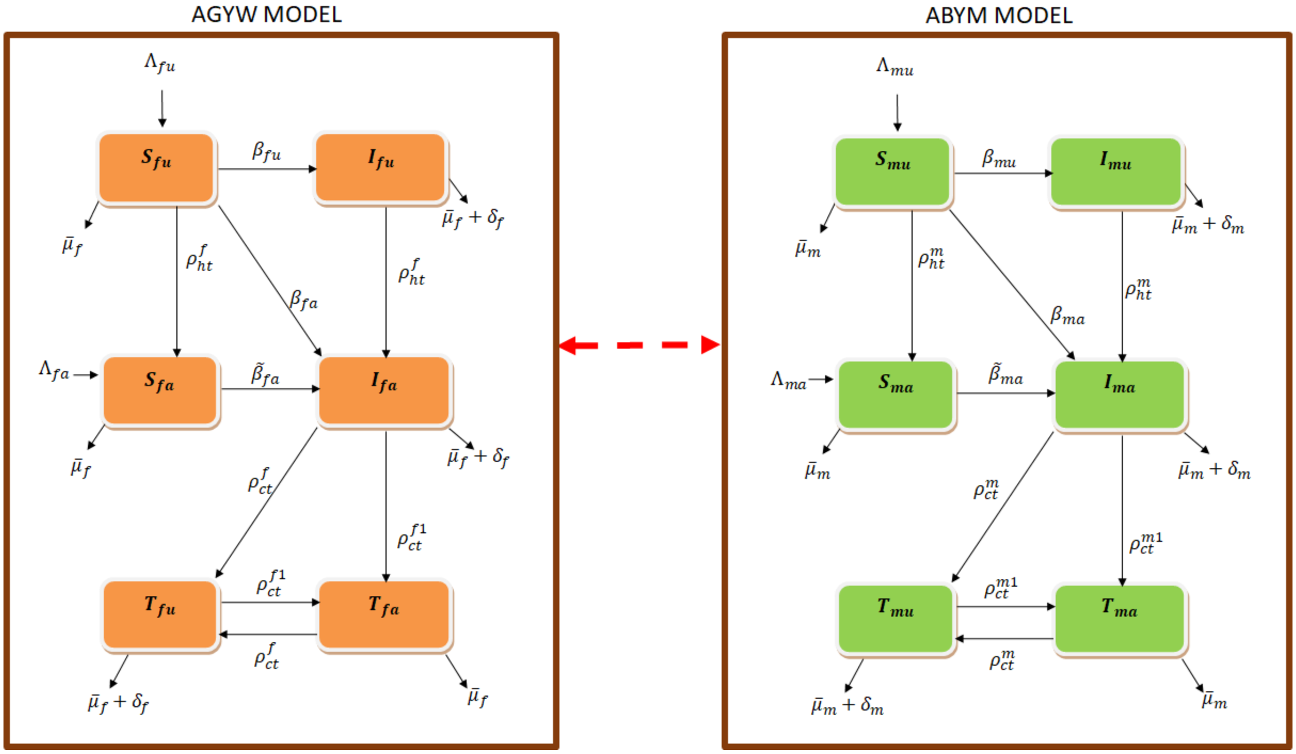

Fig 5. Schematics of the Compartmental Model. The AGYW and ABYM model describes the AGYW and ABYM transitions and interactions respectively.

The susceptibles females S_{fu}, S_{fa}, are free from the HIV infection but are at risk of infection through sexual contact with I_{mu}, I_{ma} and T_{mu} whereas the susceptibles males S_{mu}, S_{ma}, are free from the HIV infection but are at risk of infection through sexual contact with I_{fu}, I_{fa} and T_{fu}. Infectivity in I_{fu}, I_{mu} is much higher compared to I_{fa}, I_{ma} and T_{fu}, T_{mu} as the latter populations are more cautious given their infection status awareness compared to I_{fu}, I_{mu} populations. Also, T_{fu}, T_{mu} infectivity is further reduced given their partial use of condoms and ART compared to I_{fa}, I_{ma} who partially use condoms for either pregnancy or HIV/AIDS protection. Perfect adherence of T_{fa}, T_{ma} to condom use and ART reduces their viral load significantly such that they cannot sexually transmit HIV/AIDS given that undetectable viral load equals untransmittable [43]. Hence, we do not consider T_{fa}, T_{ma} populations infectious in this model as their infectivity risks are negligible. The susceptible classes S_{fu}, S_{mu} are at risk of infection at the incidence rates β_{fu}, β_{mu}, β_{fa}, β_{ma} whereas S_{fa}, S_{ma} are at risk of infection at the incidence rates $\tilde{\beta}_{fa}$, $\tilde{\beta}_{ma}$. The rates β_{fu}, β_{mu}, β_{fa}, β_{ma}, $\tilde{\beta}_{fa}$ and $\tilde{\beta}_{ma}$ are given in equations (1) as

$$
\begin{cases}
\beta_{fu} = \dfrac{c_f \gamma_f}{N_m} \left[I_{mu} + \alpha_c^m \rho_c I_{ma} + (\alpha_c^m \rho_c + \alpha_t^m \rho_t) T_{mu} \right], \\
\beta_{fa} = \dfrac{c_f \gamma_f}{N_m} \left[I_{mu} + \alpha_c^m \rho_c I_{ma} + (\alpha_c^m \rho_c + \alpha_t^m \rho_t) T_{mu} \right] \alpha_{ht}^m \rho_{ht}, \\
\tilde{\beta}_{fa} = \dfrac{c_f \gamma_f}{N_m} \left[I_{mu} + \alpha_c^m \rho_c I_{ma} + (\alpha_c^m \rho_c + \alpha_t^m \rho_t) T_{mu} \right] \alpha_{ht}^{m1} \rho_{ht}, \\
\beta_{mu} = \dfrac{c_m \gamma_m}{N_f} \left[I_{fu} + \alpha_c^f \rho_c I_{fa} + (\alpha_c^f \rho_c + \alpha_t^f \rho_t) T_{fu} \right], \\
\beta_{ma} = \dfrac{c_m \gamma_m}{N_f} \left[I_{fu} + \alpha_c^f \rho_c I_{fa} + (\alpha_c^f \rho_c + \alpha_t^f \rho_t) T_{fu} \right] \alpha_{ht}^f \rho_{ht}, \\
\tilde{\beta}_{ma} = \dfrac{c_m \gamma_m}{N_f} \left[I_{fu} + \alpha_c^f \rho_c I_{fa} + (\alpha_c^f \rho_c + \alpha_t^f \rho_t) T_{fu} \right] \alpha_{ht}^{f1} \rho_{ht}.
\end{cases} \tag{1}
$$

Contacts c_f, c_m are the average number of sexual interactions by AGYW/ABYM with individuals of the opposite sex per unit time whereas γ_f, γ_m are the probabilities of disease transmission by AGYW/ABYM with individuals of the opposite sex per unit time. Condom use rate (ρ_c) decreases the disease spread by I_{fa}, I_{ma} whereas condom use and ART adherence rate (ρ_t) reduces the infection risk by T_{fu}, T_{mu}. HIV/AIDS status disclosure (ρ_{ht}) by newly HIV/AIDS tested I_{fu}, I_{mu} and already tested populations I_{fa}, I_{ma}, T_{fu}, T_{mu} further reduces the disease spread to the susceptible populations.

When each of the HIV/AIDS controls ρ_{ht}, ρ_c, ρ_t in the AGYW/ABYM populations is 1 we have perfect adherence otherwise, $0 \leq \rho_{ht}, \rho_c, \rho_t < 1$. The rates α_{ht}^f, α_{ht}^m represent negative attitudes affecting the efficacy of HIV testing rate ρ_{ht} in the AGYW and ABYM populations such as poor health services, poverty, psycho-social conditions, socio-demographic characteristics among others [8–10]. Rates α_c^f, α_c^m represent negative attitudes affecting the efficacy of condom use rate in the AGYW and ABYM populations such as religion, peer influence, perceived individual's risk among others [3, 12–15]. Also, α_t^f, α_t^m represent negative attitudes affecting the efficacy of ART usage rate among the infected AGYW and ABYM such as stigma, poverty, caregivers waning support, confidentiality breaches by health workers among others [9,10,20,21]. Section 1 highlights how societal attitudes affect HIV/AIDS testing rates, condom use and adherence to ART among the youth in Kenya. The rates $\alpha_c^f \rho_c$, $\alpha_c^m \rho_c$ acts on I_{fa}, I_{ma} to reduce their infectivity as condom use serves to protect susceptible AGYW and ABYM from acquiring new HIV/AIDS infection. In addition to condom use, T_{fu}, T_{mu} partially uses ART which works to reduce their HIV/AIDS viral load. The combined effects of condom use and ART usage ($\alpha_c^f \rho_c + \alpha_t^f \rho_t$, $\alpha_c^m \rho_c + \alpha_t^m \rho_t$) further reduces the infectivity of T_{fu}, T_{mu} as $0 < \alpha_c^f, \alpha_c^m, \alpha_t^f, \alpha_t^m < 1$. Thus, T_{fu}, T_{mu} infectivity is less than I_{fa}, I_{ma} which is less than I_{fu}, I_{mu}.

Incidence rates by untested AGYW/ABYM with individuals of the opposite sex per unit time are given as β_{fu}, β_{mu} respectively . The incidence rates β_{fa}, β_{ma} are given by HIV/AIDS tested AGYW/ABYM but not under ART treatment with individuals of the opposite sex per unit time. The incidence rates $\tilde{\beta}_{fa}$, $\tilde{\beta}_{ma}$ results from HIV/AIDS tested AGYW/ABYM who are not perfectly adherent to consistent condom use and ART treatment with individuals of the opposite sex per unit time. The incidence rates β_{fu}, β_{mu}, β_{fa}, β_{ma}, $\tilde{\beta}_{fa}$ and $\tilde{\beta}_{ma}$ have proportionate mixing incidences since some of the adolescents and young adults aged 15-24 will have already initiated sex with most of them remaining sexually active.

Uninfected unaware S_{fu}, S_{mu} who know their HIV/AIDS status through HIV testing moves to S_{fa}, S_{ma} at the rates ρ_{ht}^{f}, ρ_{ht}^{m} with $\rho_{ht}^{f} = \alpha_{ht}^{f}\rho_{ht}$ and $\rho_{ht}^{m} = \alpha_{ht}^{m}\rho_{ht}$. A newly infected S_{fu}, S_{mu} through interaction with infected I_{mu}, I_{ma} or T_{mu} who fail to disclose their HIV/AIDS status will move to I_{fu}, I_{mu} at the rates β_{fu}, β_{mu}. Also, a newly infected S_{fu}, S_{mu} through sexual contact with infected aware populations of the opposite sex will move to I_{fa}, I_{ma} at the rates β_{fa}, β_{ma} given that status disclosure by the infected aware populations results in HIV/AIDS awareness of the newly infected S_{fu}, S_{mu}. A newly infected S_{fa}, S_{ma} moves to I_{fa}, I_{ma} at the rates $\tilde{\beta}_{fa}$, $\tilde{\beta}_{ma}$. Infected unaware I_{fu}, I_{mu} can move to I_{fa}, I_{ma} at the rates ρ_{ht}^{f}, ρ_{ht}^{m} through HIV/AIDS testing. Also, I_{fa}, I_{ma} and T_{fu}, T_{mu} who consistently use condoms and adhere to ART treatment moves to T_{fa}, T_{ma} at the rates ρ_{ct}^{f1}, ρ_{ct}^{m1} whereas an I_{fa}, I_{ma} or T_{fa}, T_{ma} who fail to use condoms consistently or adhere to ART treatment moves to T_{fu}, T_{mu} at the rates ρ_{ct}^{f}, ρ_{ct}^{m} respectively with $\rho_{ct}^{f1} = \alpha_{c}^{f1}\rho_c + \alpha_{t}^{f1}\rho_t$, $\rho_{ct}^{m1} = \alpha_{c}^{m1}\rho_c + \alpha_{t}^{m1}\rho_t$, $\rho_{ct}^{f} = \alpha_{c}^{f}\rho_c + \alpha_{t}^{f}\rho_t$ and $\rho_{ct}^{m} = \alpha_{c}^{m}\rho_c + \alpha_{t}^{m}\rho_t$ respectively. α_{ht}^{f1}, α_{ht}^{m1}, α_{c}^{f1}, α_{t}^{f1}, α_{c}^{m1}, α_{t}^{m1} and α_{ht}^{f}, α_{ht}^{m}, α_{c}^{f}, α_{t}^{f}, α_{c}^{m}, α_{t}^{m} are parameters representing negative/positive attitudes influencing HIV/AIDS controls (ρ_{ht}, ρ_c, ρ_t) but not to zero given that in the Kenyan HIV/AIDS youth dynamics some control measures are in place [52]. The rates α_{ht}^{f1}, α_{ht}^{m1}, α_{c}^{f1}, α_{t}^{f1}, α_{c}^{m1} represent attitudes affecting the efficacy of ρ_{ht}, ρ_c, ρ_t positively such as confidentiality by health workers, adequate support structure at home and the community at large, improved financial status among others whereas α_{ht}^{f}, α_{ht}^{m}, α_{c}^{f}, α_{t}^{f}, α_{c}^{m} represent negative attitudes, which was explained earlier, influencing the said controls. The rates ρ_{ct}^{f}, ρ_{ct}^{m} represent combined condom use and ART use coupled with negative attitudes whereas ρ_{ct}^{f1}, ρ_{ct}^{m1} represent combined condom use and ART use coupled with positive attitudes among the AGYW and ABYM respectively. Thus,

$$0 < \alpha_{ht}^{f1}, \alpha_{ht}^{m1}, \alpha_{c}^{f1}, \alpha_{t}^{f1}, \alpha_{c}^{m1}, \alpha_{t}^{m1}, \alpha_{ht}^{f}, \alpha_{ht}^{m}, \alpha_{c}^{f}, \alpha_{t}^{f}, \alpha_{c}^{m}, \alpha_{t}^{m} < 1$$

with

$$\alpha_{ht}^{f1}, \alpha_{ht}^{m1}, \alpha_{c}^{f1}, \alpha_{t}^{f1}, \alpha_{c}^{m1}, \alpha_{t}^{m1} > \alpha_{ht}^{f}, \alpha_{ht}^{m}, \alpha_{c}^{f}, \alpha_{t}^{f}, \alpha_{c}^{m}, \alpha_{t}^{m}.$$

Recruitment rates into susceptible populations S_{fu}, S_{mu}, S_{fa}, S_{ma} is by natural births and maturity to 15 years and are given as Λ_{fu}, Λ_{mu}, Λ_{fa}, Λ_{ma} respectively. The susceptible classes are all reduced by natural deaths μ_f, μ_m whereas the infectious classes are all decreased by natural deaths and disease induced deaths, δ_f, δ_m. Upon turning 24 years, the AGYW and the ABYM population exit the model at the rate σ. The state variables and parameters are assumed to be positive given that a population dynamics model is being studied. Tables 1 and 2 gives the summary description for the state variables and model parameters respectively.

The system of ordinary differential equations governing the AGYW/ABYM HIV model is given by the system of equations (2) as

$$
\begin{cases}
\dfrac{dS_{fu}}{dt} = \Lambda_{fu} - \beta_{fu}\,S_{fu} - \beta_{fa}\,S_{fu} - \mu_{f1}\,S_{fu},\\
\dfrac{dS_{fa}}{dt} = \Lambda_{fa} + \rho^{f}_{ht}\,S_{fu} - \tilde{\beta}_{fa}\,S_{fa} - \mu_{f2}\,S_{fa},\\
\dfrac{dI_{fu}}{dt} = \beta_{fu}\,S_{fu} - \mu_{f3}\,I_{fu},\\
\dfrac{dI_{fa}}{dt} = \tilde{\beta}_{fa}\,S_{fa} + \beta_{fa}\,S_{fu} + \rho^{f}_{ht}\,I_{fu} - \mu_{f4}\,I_{fa},\\
\dfrac{dT_{fu}}{dt} = \rho^{f}_{ct}\,I_{fa} + \rho^{f}_{ct}\,T_{fa} - \mu_{f5}\,T_{fu},\\
\dfrac{dT_{fa}}{dt} = \rho^{f1}_{ct}\,I_{fa} + \rho^{f1}_{ct}\,T_{fu} - \mu_{f6}\,T_{fa},\\
\dfrac{dS_{mu}}{dt} = \Lambda_{mu} - \beta_{mu}\,S_{mu} - \beta_{ma}\,S_{mu} - \mu_{m1}\,S_{mu},\\
\dfrac{dS_{ma}}{dt} = \Lambda_{ma} + \rho^{m}_{ht}\,S_{mu} - \tilde{\beta}_{ma}\,S_{ma} - \mu_{m2}\,S_{ma},\\
\dfrac{dI_{mu}}{dt} = \beta_{mu}\,S_{mu} - \mu_{m3}\,I_{mu},\\
\dfrac{dI_{ma}}{dt} = \tilde{\beta}_{ma}\,S_{ma} + \beta_{ma}\,S_{mu} + \rho^{m}_{ht}\,I_{mu} - \mu_{m4}\,I_{ma},\\
\dfrac{dT_{mu}}{dt} = \rho^{m}_{ct}\,I_{ma} + \rho^{m}_{ct}\,T_{ma} - \mu_{m5}\,T_{mu},\\
\dfrac{dT_{ma}}{dt} = \rho^{m1}_{ct}\,I_{ma} + \rho^{m1}_{ct}\,T_{mu} - \mu_{m6}\,T_{ma}.
\end{cases}
\tag{2}
$$

where 253
$\bar{\mu}_f = \mu_f + \sigma$, $\mu_{f1} = \rho^{f}_{ht} + \bar{\mu}_f$, $\mu_{f2} = \bar{\mu}_f$, $\mu_{f3} = \rho^{f}_{ht} + \bar{\mu}_f + \delta_f$, $\mu_{f4} = \rho^{f}_{ct} + \rho^{f1}_{ct} + \bar{\mu}_f +$ 254
δ_f, $\mu_{f5} = \rho^{f1}_{ct} + \bar{\mu}_f + \delta_f$, $\mu_{f6} = \rho^{f}_{ct} + \bar{\mu}_f$, $\bar{\mu}_m = \mu_m + \sigma$, $\mu_{m1} = \rho^{m}_{ht} + \bar{\mu}_m$, $\mu_{m2} = \bar{\mu}_m$, $\mu_{m3} =$ 255
$\rho^{m}_{ht} + \bar{\mu}_m + \delta_m$, $\mu_{m4} = \rho^{m}_{ct} + \rho^{m1}_{ct} + \bar{\mu}_m + \delta_m$, $\mu_{m5} = \rho^{m1}_{ct} + \bar{\mu}_m + \delta_m$, $\mu_{m6} = \rho^{m}_{ct} + \bar{\mu}_m$. 256

Table 1. Summary Description of State variables

Variable	Description
S_{fu}, S_{mu}	Susceptible AGYW & ABYM who have never tested for HIV/AIDS
S_{fa}, S_{ma}	Susceptible AGYW & ABYM who have ever tested for HIV/AIDS
I_{fu}, I_{mu}	Infected AGYW & ABYM who have never tested for HIV/AIDS
I_{fa}, I_{ma}	Infected AGYW & ABYM who have ever tested for HIV/AIDS
T_{fu}, T_{mu}	Infected aware AGYW & ABYM who are not adherent to ART or consistent condom use
T_{fa}, T_{ma}	Infected aware AGYW & ABYM who are adherent to ART and use condoms consistently

Table 2. Summary Description of Parameters

Parameter	Description
Λ_{fu}, Λ_{mu}	Natural birth and maturity rates of susceptible AGYW and ABYM unaware of their HIV status
Λ_{fa}, Λ_{ma}	Natural birth and maturity rates of susceptible AGYW and ABYM aware of their HIV status
ρ_{ht}	AGYW/ABYM HIV/AIDS testing rate
ρ_t	AGYW/ABYM adherence rate to anti-retroviral therapy treatment
ρ_c	AGYW/ABYM condom use rate
μ_f, μ_m	Natural death rates of AGYW and ABYM respectively
γ_f, γ_m	Probabilities of AGYW and ABYM transmission risk
δ_f, δ_m	Disease induced deaths in AGYW and ABYM respectively
c_f, c_m	AGYW and ABYM sexual contact rates
α_{ht}^{f}, α_{ht}^{m}, α_{ht}^{f1}, α_{ht}^{m1}	Negative and positive attitude rates influencing HIV/AIDS testing rates among the AGYW and ABYM respectively
α_{c}^{f}, α_{c}^{m}, α_{c}^{f1}, α_{c}^{m1}	Negative and positive attitude rates influencing condom use rates among the AGYW and ABYM respectively
α_{t}^{f}, α_{t}^{m}, α_{t}^{f1}, α_{t}^{m1}	Negative and positive attitude rates influencing ART adherence rates among the AGYW and ABYM respectively
σ	Exit rate of AGYW and ABYM upon turning 24 years

2.3 Model Properties

Mathematical analysis of the formulated model system (2) is presented here. We show that the compact system of ordinary differential equations (2) governing the model of biological interest is well-posed and control reproduction number with its biological interpretation given. The conditions for stability of the model steady states are determined.

2.3.1 Boundedness

Theorem 2.1 *The model* (2) *solutions are uniformly bounded in a set*

$$\Omega = \left\{ (S_{fu},\, S_{fa},\, I_{fu},\, I_{fa},\, T_{fu},\, T_{fa},\, S_{mu},\, S_{ma},\, I_{mu},\, I_{ma},\, T_{mu},\, T_{ma}) \in \mathbb{R}_{12}^{+} \;|N(0) \leq N \leq \frac{\tilde{\Lambda}}{\mu_f + \mu_m} \right\}.$$

Proof 2.1 *Given that system* (2) *is a finite dimensional dynamical system, its initial conditions and boundary conditions need to be constrained to* Ω. *Let* $(S_{fu}, S_{fa}, I_{fu}, I_{fa}, T_{fu}, T_{fa}, S_{mu}, S_{ma}, I_{mu}, I_{ma}, T_{mu}, T_{ma})$ *be the solution to* (2) *and* $S_{fu}(0) = S_{fu}^0 \geq 0$, $S_{fa}(0) = S_{fa}^0 \geq 0$, $I_{fu}(0) = I_{fu}^0 \geq 0$, $I_{fa}(0) = I_{fa}^0 \geq 0$, $T_{fu}(0) = T_{fu}^0 \geq 0$, $T_{fa}(0) = T_{fa}^0 \geq 0$ $S_{mu}(0) = S_{mu}^0 \geq 0$, $S_{ma}(0) = S_{ma}^0 \geq 0$, $I_{mu}(0) = I_{mu}^0 \geq 0$, $I_{ma}(0) = I_{ma}^0 \geq 0$, $T_{mu}(0) = T_{mu}^0 \geq 0$, $T_{ma}(0) = T_{ma}^0 \geq 0$ *be the initial conditions. Adding all equations of system* (2), *yields*

$$\begin{aligned} \dot{N} &= (\tilde{\Lambda}) - \bar{\mu}_f\, N_f - \bar{\mu}_m\, N_m - \delta_f\, (N_f - \tilde{N}_f) - \delta_m\, (N_m - \tilde{N}_m) \\ &\leq \tilde{\Lambda} - (\bar{\mu}_f + \delta_f) N_f - (\bar{\mu}_m + \delta_m) N_m - \delta_f \tilde{N}_f - \delta_m \tilde{N}_m \\ &\leq \tilde{\Lambda} - \tilde{\mu} N \end{aligned}$$

where $\tilde{\Lambda} = \Lambda_{fu} + \Lambda_{fa} + \Lambda_{mu} + \Lambda_{ma}$, $\tilde{N}_f = S_{fu} + S_{fa} + T_{fa}$, $\tilde{N}_m = S_{mu} + S_{ma} + T_{ma}$, $\tilde{\mu} = min(\bar{\mu}_f + \delta_f,\, \bar{\mu}_m + \delta_m)$. *Thus,* Ω *is a compact attracting non-negatively invariant for positive starting-point values since* $N(0) > 0$. *This can easily be proved using the theory*

of differential inequality [44]. All solutions of (2) *originating in* $\mathbb{R}^{12}_+$ *are confined in* Ω. *Let* M *be an upper bound for* S_{fu}, S_{fa}, I_{fu}, I_{fa}, T_{fu}, T_{fa}, S_{mu}, S_{ma}, I_{mu}, I_{ma}, T_{mu}, T_{ma}. *We then conclude that every solution originating from* Ω *stays in* Ω *and is bounded by* M.

2.3.2 Local existence and uniqueness

Lemma 2.1 *Let* $x = (x_i)_{i=1,2,\dots,12}$ *and* $f : \mathbb{R}_+ \times \mathbb{R}^{12} \to \mathbb{R}^{12}$ *be continuous with respect to* t, x *and Lipschitz continuous. Let* $f(t, x)$ *be non negative for all* $(t, x) \in \mathbb{R}_+ \times \mathbb{R}^{12}$, *and* $x_i = 0$. *For every* $x_0 \in \mathbb{R}^{12}_+$, *there exists a positive constant* T *such that* $\dot{x} = f(t, x), \quad x(t_0) = x_0$, *has a unique, positive and existing solution whose value lies in the interval* $[0, T)$ *and in* $\mathbb{R}^{12}_+$. *If* $T < \infty$ *then* $\limsup_{t\to T} \sum_{i=1}^{12} x_i = +\infty$.

Theorem 2.2 *The solution set* $\{S_{fu}, S_{fa}, I_{fu}, I_{fa}, T_{fu}, T_{fa}, S_{mu}, S_{ma}, I_{mu}, I_{ma}, T_{mu}, T_{ma}\}$ *of the model* (2) *exists, is unique and positive for* $t > 0$.

By theorem 2.1, the solutions to (2) are uniformly bounded on $[0, T)$. By theorem 2.2, the solution of (2) exists for any finite time. Thus, for any positive initial data in $\mathbb{R}^{12}_+$, the model system (2) will possess a unique and positive solution in $\mathbb{R}^{12}_+$. This proves that all feasible solution of the model system (2) lies in the feasible region, Ω.

2.3.3 Equilibria

The model system (2) has a unique disease-free equilibrium (DFE)

$$E^0 = (S^0_{fu}, S^0_{fa}, 0, 0, 0, 0, S^0_{mu}, S^0_{ma}, 0, 0, 0, 0)$$

and possibly an endemic equilibrium (EE)

$$E^* = (S^*_{fu}, S^*_{fa}, I^*_{fu}, I^*_{fa}, T^*_{fu}, T^*_{fa}, S^*_{mu}, S^*_{ma}, I^*_{mu}, I^*_{ma}, T^*_{mu}, T^*_{ma})$$

with

$$\begin{cases}
S^0_{fu} = \dfrac{\Lambda_{fu}}{\mu_{f1}}, \quad S^0_{fa} = \dfrac{\Lambda_{fa}\,\mu_{f1} + \rho^f_{ht}\Lambda_{fu}}{\mu_{f1}\,\mu_{f2}}, \\
S^0_{mu} = \dfrac{\Lambda_{mu}}{\mu_{m1}}, \quad S^0_{ma} = \dfrac{\Lambda_{ma}\,\mu_{m1} + \rho^m_{ht}\Lambda_{mu}}{\mu_{m1}\,\mu_{m2}}, \\
S^*_{fu} = \dfrac{\Lambda_{fu}}{g_{02}\beta^*_{fu} + \mu_{f1}}, \; S^*_{fa} = \dfrac{\Lambda_{fa}}{\rho^{m1}_{ht}\beta_{fu}* + \mu_{f2}} + \dfrac{\rho^f_{ht}\,\Lambda_{fu}}{(\rho^{m1}_{ht}\beta^*_{fu} + \mu_{f2})(g_{02}\,\beta^*_{fu} + \mu_{f1})}, \\
I^*_{fu} = \dfrac{\Lambda_{fu}\,\beta^*_{fu}}{\mu_{f3}\,(g_{02}\beta^*_{fu} + \mu_{f1})}, \; I^*_{fa} = \dfrac{q_{02}\,\beta^{*2}_{fu} + q_{03}\,\beta^*_{fu} + q_{04}}{q_{05}\,\beta^{*2}_{fu} + q_{06}\,\beta^*_{fu} + q_{07}}, \; T^*_{fu} = g_{01}\,I^*_{fa}, \; T^*_{fa} = g_{00}\,I^*_{fa}, \\
S^*_{mu} = \dfrac{\Lambda_{mu}}{g_{08}\beta^*_{mu} + \mu_{m1}}, \; S^*_{ma} = \dfrac{\Lambda_{ma}}{\rho^{f1}_{ht}\beta_{mu}* + \mu_{m2}} + \dfrac{\rho^m_{ht}\,\Lambda_{mu}}{(\rho^{f1}_{ht}\beta^*_{mu} + \mu_{m2})(g_{08}\,\beta^*_{mu} + \mu_{m1})}, \\
I^*_{mu} = \dfrac{\Lambda_{mu}\,\beta^*_{mu}}{\mu_{m3}\,(g_{08}\beta^*_{mu} + \mu_{m1})}, \; I^*_{ma} = \dfrac{h_{02}\,\beta^{*2}_{mu} + h_{03}\,\beta^*_{mu} + h_{04}}{h_{05}\,\beta^{*2}_{mu} + h_{06}\,\beta^*_{mu} + h_{07}}, \; T^*_{mu} = g_{07}\,I^*_{ma}, \\
T^*_{ma} = g_{06}\,I^*_{ma}, \; N^*_f = \dfrac{\Lambda_{fu} + \Lambda_{fa} + \delta_f\,\tilde{N}^*_f}{\bar{\mu}_f + \delta_f}, \; \tilde{N}^*_f = S^*_{fu} + S^*_{fa} + T^*_{fa}, \\
N^*_m = \dfrac{\Lambda_{mu} + \Lambda_{ma} + \delta_m\,\tilde{N}^*_m}{\bar{\mu}_m + \delta_m}, \; \tilde{N}^*_m = S^*_{mu} + S^*_{ma} + T^*_{ma}, \\
\beta^{*5}_{fu} + C_1\,\beta^{*4}_{fu} + C_2\,\beta^{*3}_{fu} + C_3\,\beta^{*2}_{fu} + C_4\,\beta^*_{fu} - C_5 = 0, \\
\beta^{*5}_{mu} + C_{11}\,\beta^{*4}_{mu} + C_{21}\,\beta^{*3}_{mu} + C_{31}\,\beta^{*2}_{mu} + C_{41}\,\beta^*_{mu} - C_{51} = 0.
\end{cases} \tag{3}$$

Refer to appendix 15 for the expressions of g_{00}, $g_{01}, ..., g_{11}$, q_{01}, $q_{02}, ..., q_{20}$, h_{01}, $h_{02}, ..., h_{20}$, C_1, $C_2, ..., C_5$ and C_{11}, $C_{21}, ..., C_{51}$.

By the fundamental theorem of algebra, the polynomial equations $\beta_{fu}^{*5} + C_1\,\beta_{fu}^{*4} + C_2\,\beta_{fu}^{*3} + C_3\,\beta_{fu}^{*2} + C_4\,\beta_{fu}^{*} - C_5 = 0$ and $\beta_{mu}^{*5} + C_{11}\,\beta_{mu}^{*4} + C_{21}\,\beta_{mu}^{*3} + C_{31}\,\beta_{mu}^{*2} + C_{41}\,\beta_{mu}^{*} - C_{51} = 0$, of odd degree, have at least one real root each. By Descartes' rule of signs, the polynomial equations will each have at least one non-negative real root if and only if $C_1 > 0$, $C_2 > 0$, $C_3 > 0$, $C_4 > 0$, $C_5 > 0$ and $C_{11} > 0$, $C_{21} > 0$, $C_{31} > 0$, $C_{41} > 0$, $C_{51} > 0$, given that the sign before C_5 and C_{51} is negative and the sign before β_{fu}^{*5} and β_{mu}^{*5} is non-negative otherwise each of the polynomial equation will have at most four (4) non-negative real roots. The exact number of non-negative roots can be determined using Descartes' rule of signs and Euclid's algorithm of the Sturm's theorem.

2.4 Control Reproduction Number, $\mathcal{R}_c$

The control reproduction number, $\mathcal{R}_c$, is defined as the expected number of secondary infections produced by a typical infected individual during its entire period of infectiousness in a population that is not entirely susceptible due to the presence of control efforts [45]. The controls present in our model are HIV/AIDS testing (ρ_{ht}), condom use (ρ_c) and ART adherence (ρ_t).

The global dynamics for many disease models is determined by the sharp threshold criterion given by the basic reproduction number and this is true for our model system (2) [46]. Model system (2) possesses a sharp threshold if the control reproduction number $\mathcal{R}_c$ given by equation 7 is such that E^0 is globally attractive for $\mathcal{R}_c \leq 1$ and there is a unique endemic equilibrium E^* that is globally attractive in the feasible region for $\mathcal{R}_c > 1$. Biologically, $\mathcal{R}_c$ is used to measure the transmission potential of the HIV/AIDS disease among the AGYW and ABYM in the presence of the said controls [46]. The threshold property states that if $\mathcal{R}_c > 1$, HIV/AIDS disease persists in the youthful population hence becoming endemic whereas when $\mathcal{R}_c < 1$, the disease mirrors the effects of successful combinatory control efforts to the AGYW and ABYM consequently protecting the susceptible youth from acquiring new HIV/AIDS infection.

The next generation matrix approach is used to compute the control reproduction number for the model system (2) [46]. Consider the infected subsystem of the model system (2) given as

$$
\begin{cases}
\dfrac{dI_{fu}}{dt} = \beta_{fu}\,S_{fu} - \mu_{f3}\,I_{fu},\\
\dfrac{dI_{fa}}{dt} = \tilde{\beta}_{fa}\,S_{fa} + \beta_{fa}\,S_{fu} + \rho_{ht}^{f}\,I_{fu} - \mu_{f4}\,I_{fa},\\
\dfrac{dT_{fu}}{dt} = \rho_{ct}^{f}\,I_{fa} + \rho_{ct}^{f}\,T_{fa} - \mu_{f5}\,T_{fu},\\
\dfrac{dI_{mu}}{dt} = \beta_{mu}\,S_{mu} - \mu_{m3}\,I_{mu},\\
\dfrac{dI_{ma}}{dt} = \tilde{\beta}_{ma}\,S_{ma} + \beta_{ma}\,S_{mu} + \rho_{ht}^{m}\,I_{mu} - \mu_{m4}\,I_{ma},\\
\dfrac{dT_{mu}}{dt} = \rho_{ct}^{m}\,I_{ma} + \rho_{ct}^{m}\,T_{ma} - \mu_{m5}\,T_{mu}.
\end{cases}
\tag{4}
$$

The right hand side of the infected subsystem (4) is decomposed into two parts, F and V where F denotes the transmission part and each F_i represents new infection. V denotes

the transition part and each V_i describes change in state for instance removal through natural deaths, disease induced deaths, aging, HIV/AIDS status knowledge, condom use and ART adherence [47].

$$F = \begin{bmatrix} \left(\frac{c_f\gamma_f}{N_m}\left[I_{mu} + \alpha_c^m\rho_c^m I_{ma} + (\alpha_c^m\rho_c^m + \alpha_t^m\rho_t^m)T_{mu}\right]\right) S_{fu} \\ \rho_{ht}^m\left(\frac{c_f\gamma_f}{N_m}\left[I_{mu} + \alpha_c^m\rho_c^m I_{ma} + (\alpha_c^m\rho_c^m + \alpha_t^m\rho_t^m)T_{mu}\right]\right)(S_{fu} + \alpha_{ht}^m S_{fa}) \\ 0 \\ \left(\frac{c_m\gamma_m}{N_f}\left[I_{fu} + \alpha_c^f\rho_c^f I_{fa} + (\alpha_c^f\rho_c^f + \alpha_t^f\rho_t^f)T_{fu}\right]\right) S_{mu} \\ \rho_{ht}^f\left(\frac{c_m\gamma_m}{N_f}\left[I_{fu} + \alpha_c^f\rho_c^f I_{fa} + (\alpha_c^f\rho_c^f + \alpha_t^f\rho_t^f)T_{fu}\right]\right)(S_{mu} + \alpha_{ht}^f S_{ma}) \\ 0 \end{bmatrix}$$

and

$$V = -\begin{bmatrix} -\mu_{f3}\, I_{fu} \\ \rho_{ht}^f\, I_{fu} - \mu_{f4}\, I_{fa} \\ \rho_{ct}^f\, I_{fa} + \rho_{ct}^f\, T_{fa} - \mu_{f5}\, T_{fu} \\ -\mu_{m3}\, I_{mu} \\ \rho_{ht}^m\, I_{mu} - \mu_{m4}\, I_{ma} \\ \rho_{ct}^m\, I_{ma} + \rho_{ct}^m\, T_{ma} - \mu_{m5}\, T_{mu} \end{bmatrix}.$$

$\mathcal{F}$ and $\mathcal{V}$ are computed as:

$$\mathcal{F} = \left[\frac{\partial F_i(x_0)}{\partial x_j}\right] \; and \; \mathcal{V} = \left[\frac{\partial V_i(x_0)}{\partial x_j}\right] \tag{5}$$

where x_0 is the disease free state. Evaluating $\mathcal{F}\mathcal{V}^{-1}$ yields the next generation matrix for the model system (2) whose largest non-negative eigenvalue is the reproduction number, $\mathcal{R}_c$. $\mathcal{F}\mathcal{V}^{-1}$ and $\mathcal{R}_c$ are given as follows:

$$\mathcal{F}\mathcal{V}^{-1} = \begin{bmatrix} 0 & 0 & 0 & \omega_1\,\eta_1 & \omega_1\,\eta_2 & \omega_1\,\eta_3 \\ 0 & 0 & 0 & \omega_2\,\eta_1 & \omega_2\,\eta_2 & \omega_2\,\eta_3 \\ 0 & 0 & 0 & 0 & 0 & 0 \\ \omega_3\,\varepsilon_1 & \omega_3\,\varepsilon_2 & \omega_3\,\varepsilon_3 & 0 & 0 & 0 \\ \omega_4\,\varepsilon_1 & \omega_4\,\varepsilon_2 & \omega_4\,\varepsilon_3 & 0 & 0 & 0 \\ 0 & 0 & 0 & 0 & 0 & 0 \end{bmatrix}, \tag{6}$$

$$\mathcal{R}_c = \sqrt{\mathcal{R}_{uf}\,\mathcal{R}_{um} + \mathcal{R}_{af}\,\mathcal{R}_{am} + \mathcal{R}_{uf}\,\mathcal{R}_{am} + \mathcal{R}_{af}\,\mathcal{R}_{um}} \tag{7}$$

with

$$
\begin{cases}
\mathcal{R}_{uf} = \omega_1\,\epsilon_1, \quad \mathcal{R}_{um} = \omega_3\,\eta_1, \quad \mathcal{R}_{af} = \omega_2\,\epsilon_2, \quad \mathcal{R}_{am} = \omega_4\,\eta_2, \\
\mathcal{R}_u = \mathcal{R}_{uf}\,\mathcal{R}_{um}, \quad \mathcal{R}_a = \mathcal{R}_{af}\,\mathcal{R}_{am}, \quad \mathcal{R}_{mm} = \mathcal{R}_{uf}\,\mathcal{R}_{am}, \quad \mathcal{R}_{mf} = \mathcal{R}_{af}\,\mathcal{R}_{um}, \\
\omega_1 = \dfrac{c_f\,\gamma_f\,S^0_{fu}}{S^0_{mu} + S^0_{ma}}, \quad \omega_2 = \dfrac{\rho^m_{ht}\,c_f\,\gamma_f\,(S^0_{fu} + \alpha^m_{ht}\,S^0_{fa})}{S^0_{mu} + S^0_{ma}}, \\
\omega_3 = \dfrac{c_m\,\gamma_m\,S^0_{mu}}{S^0_{fu} + S^0_{fa}}, \quad \omega_4 = \dfrac{\rho^f_{ht}\,c_m\,\gamma_m\,(S^0_{mu} + \alpha^f_{ht}\,S^0_{ma})}{S^0_{fu} + S^0_{fa}}, \\
\eta_1 = \dfrac{1}{\mu_{m3}} + \dfrac{\alpha^m_c\,\rho_c\rho^m_{ht}}{\mu_{m3}\,\mu_{m4}} + \dfrac{(\alpha^m_c\,\rho_c + \alpha^m_t\,\rho_t)\,\rho^m_{ct}\rho^m_{ht}}{\mu_{m3}\,\mu_{m4}\,\mu_{m5}}, \\
\eta_2 = \dfrac{\alpha^m_c\,\rho_c}{\mu_{m4}} + \dfrac{(\alpha^m_c\,\rho_c + \alpha^m_t\,\rho_t)\,\rho^m_{ct}}{\mu_{m4}\,\mu_{m5}}, \quad \eta_3 = \dfrac{(\alpha^m_c\,\rho_c + \alpha^m_t\,\rho_t)}{\mu_{m5}}, \\
\varepsilon_1 = \dfrac{1}{\mu_{f3}} + \dfrac{\alpha^f_c\,\rho_c\rho^f_{ht}}{\mu_{f3}\,\mu_{f4}} + \dfrac{\left(\alpha^f_c\,\rho_c + \alpha^f_t\,\rho_t\right)\,\rho^f_{ct}\rho^f_{ht}}{\mu_{f4}\,\mu_{f3}\,\mu_{f5}}, \\
\varepsilon_2 = \dfrac{\alpha^f_c\,\rho_c}{\mu_{f4}} + \dfrac{\left(\alpha^f_c\,\rho_c + \alpha^f_t\,\rho_t\right)\,\rho^f_{ct}}{\mu_{f4}\,\mu_{f5}}, \quad \varepsilon_3 = \dfrac{\left(\alpha^f_c\,\rho_c + \alpha^f_t\,\rho_t\right)}{\mu_{f5}}.
\end{cases}
\tag{8}
$$

$\mathcal{R}_{uf}$, $\mathcal{R}_{um}$ gives the average number of the newly infected unaware AGYW and ABYM whereas $\mathcal{R}_{af}$, $\mathcal{R}_{am}$ gives the average number of the newly infected aware AGYW and ABYM. Newly infected youth generated by individuals with same status is given by $\mathcal{R}_{uf}\,\mathcal{R}_{um}$ and $\mathcal{R}_{af}\,\mathcal{R}_{am}$ whereas newly infected youth generated by mixed status interaction is given by $\mathcal{R}_{uf}\,\mathcal{R}_{am}$ and $\mathcal{R}_{af}\,\mathcal{R}_{um}$. In the absence of HIV/AIDS testing, condom use and ART control, the control reproduction number $\mathcal{R}_c$ reduces to the basic reproduction number $\mathcal{R}_0$ and this is given as:

$$
\mathcal{R}_0 = \sqrt{\mathcal{R}_{0f}\,\mathcal{R}_{0m}} \tag{9}
$$

with

$$
\mathcal{R}_{0f} = \frac{c_f\,\gamma_f\,S^0_{fu}}{\mu_{f3}\,(S^0_{mu} + S^0_{ma})} \quad \text{and} \quad \mathcal{R}_{0m} = \frac{c_m\,\gamma_m\,S^0_{mu}}{\mu_{m3}(S^0_{fu} + S^0_{fa})}.
$$

Using the parameter estimates for our model system given in table 6, 7 and 8, $\mathcal{R}_0$ is estimated at 20.4409 with $\mathcal{R}_{0f} = 22.9550$ and $\mathcal{R}_{0m} = 18.2021$. $\mathcal{R}_{0f} > \mathcal{R}_{0m}$ implies that the adolescent girls and young women have a greater susceptibility to HIV/AIDS infection compared to their male counterparts which is consistent with Kenyan youth HIV/AIDS disease dynamics [1]. The Kenyan reproduction number $\mathcal{R}_0$ was derived from early prevalence antenatal clinic data which was estimated at 6.34 [48]. The presence of combinatory control efforts, however low, has played a key role in reducing new HIV infections among the youthful population with our model control reproduction number $\mathcal{R}_c$ estimated at 4.1003 when $\rho_{ht} = 0.48$, $\rho_c = 0.3$ and $\rho_t = 0.1$ and control attitude rates for the low control simulations given in table 7.

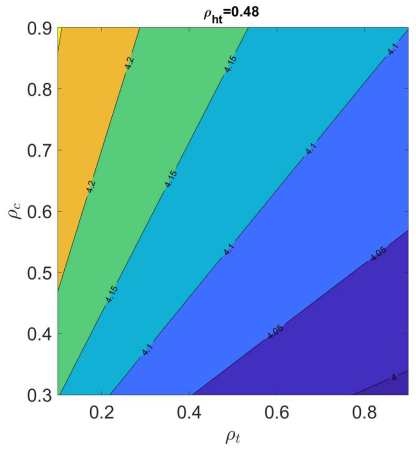

(a) Change in $\mathcal{R}_c$ with low ρ_{ht} and varying ρ_c and ρ_t

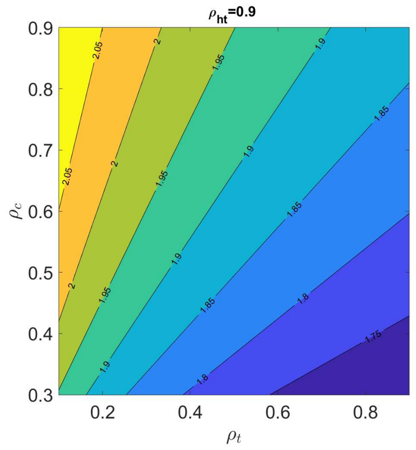

(b) Change in $\mathcal{R}_c$ with high ρ_{ht} and varying ρ_c and ρ_t

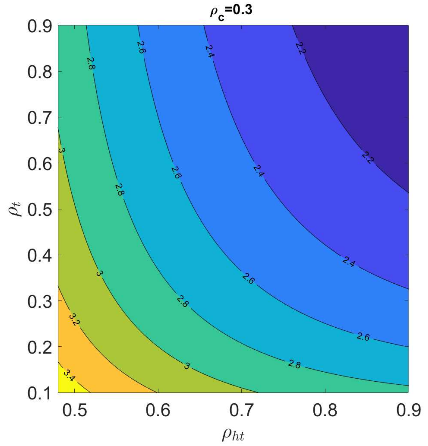

(c) Change in $\mathcal{R}_c$ with low ρ_c and varying ρ_{ht} and ρ_t

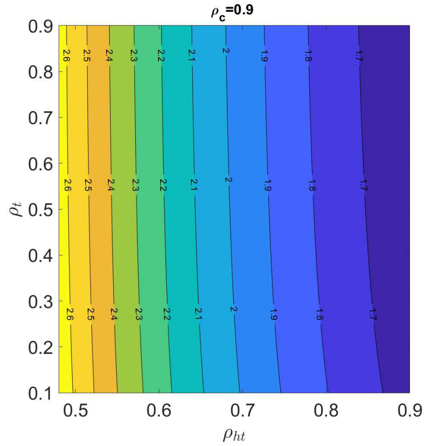

(d) Change in $\mathcal{R}_c$ with high ρ_c and varying ρ_{ht} and ρ_t

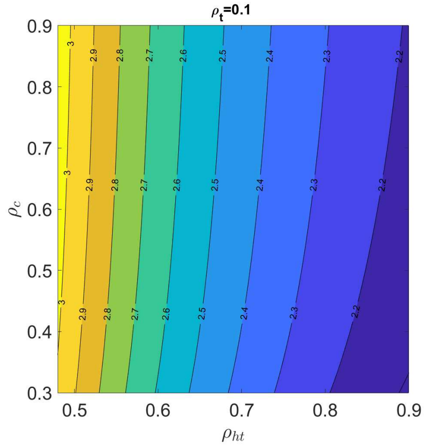

(e) Change in $\mathcal{R}_c$ with low ρ_t and varying ρ_{ht} and ρ_c

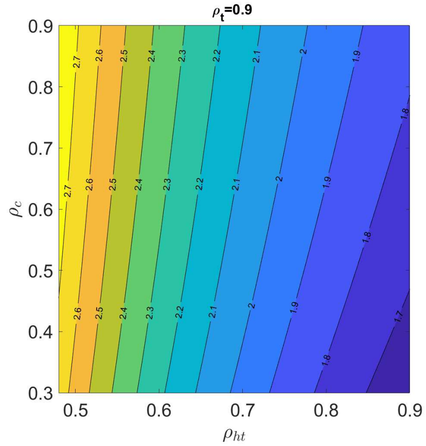

(f) Change in $\mathcal{R}_c$ with high ρ_t and varying ρ_{ht} and ρ_c

Fig 6. Change in the local control reproduction number $\mathcal{R}_c$ with varying ρ_{ht}, ρ_c and ρ_t.

Figures 6(a) -6(f)) show the change in control reproduction number with fixed HIV/AIDS controls and varying HIV/AIDS controls. The controls are varied from an estimated baseline rate to a 90% efficacy rate. Figures 6(a) -6(b)) show the change in the local control reproduction number when HIV/AIDS testing is fixed at 0.48 and 0.9 respectively while condom use and ART adherence rates are varied from $0.3-0.9$ and $0.1-0.9$ efficacy rates. Similarly, figures 6(c) -6(d)) show the change in the local control reproduction number when condom use rate is fixed at 0.3 and 0.9 respectively while HIV/AIDS testing and ART adherence rates are varied from $0.48 - 0.9$ and $0.1 - 0.9$ efficacy rates. Figures 6(e) -6(f)) show the change in the local control reproduction number when ART adherence is fixed at 0.1 and 0.9 respectively while HIV testing and condom use rates are varied from $0.48 - 0.9$ and $0.3 - 0.9$ efficacy rates.

Figures 6(b), 6(d) and 6(f) generally reflect the impact of reduced transmission potential of the control reproduction number when fixed controls are at a high efficacy rate of 0.9. The greatest reduction in the control reproduction number is realized when HIV testing rate is fixed at 0.9 with condom use and ART adherence rates increasing from their respective baseline values to 0.9 efficacy rate (see figure 6(b)). This suggests that fixed higher HIV testing rates in all populations coupled with increased condom use and ART adherence rates work well to reduce the control reproduction number but not below unity for the Kenyan youth. This implies that the current sexual interactions among the various states will sustain the HIV epidemic even when efficacy rate of 90% is achieved.

Taking the best scenario of reduced transmission potential of the control reproduction number described earlier, we unpack the unitary contributors to the control reproduction number to find the best case scenarios that could significantly reduce the control reproduction number (see figure 7). $\mathcal{R}_u$ contribution will sustain HIV/AIDS at endemic levels among the Kenyan youth population whereas $\mathcal{R}_a$ contribution will result in significant disease reduction among the AGYW and ABYM populations (see figures 7(a), 7(b)). Further, any interaction between aware male/female youth with unaware male/female youth yields good result that could lead to significant disease reduction among the Kenyan youth (see figures 7(c), 7(d)). Mixed status sexual interaction brings the control reproduction number down in our model as a result of HIV/AIDS status disclosure by the aware AGYW/ABYM. Any sexual relationship fostered with HIV/AIDS tested youth using condoms and adherent to ART promises hope for new HIV/AIDS infection reduction among the Kenyan youth.

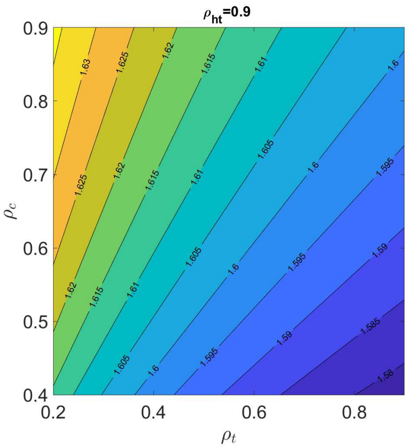

(a) Change in $\mathcal{R}_u$ with high ρ_{ht} and varying ρ_c and ρ_t

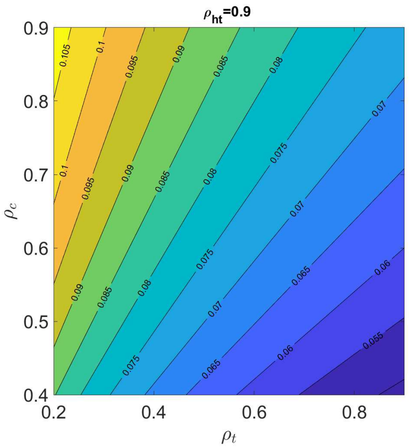

(b) Change in $\mathcal{R}_a$ with high ρ_{ht} and varying ρ_c and ρ_t

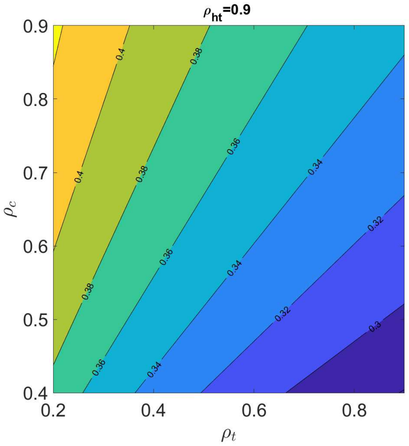

(c) Change in $\mathcal{R}_{mf}$ with high ρ_{ht} and varying ρ_c and ρ_t

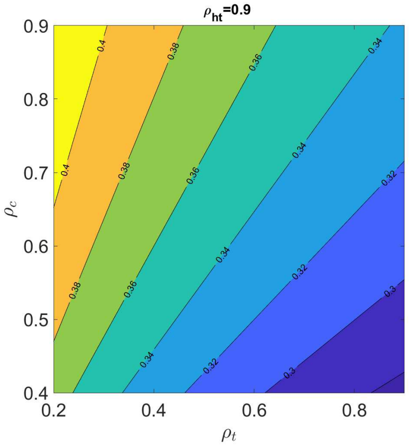

(d) Change in $\mathcal{R}_{mm}$ with high ρ_{ht} and varying ρ_c and ρ_t

Fig 7. Change in $\mathcal{R}_u$,$\mathcal{R}_a$, $\mathcal{R}_{mf}$ and $\mathcal{R}_{mm}$ with fixed $\rho_{ht} = 0.9$ and varying ρ_c and ρ_t.

2.5 Data Fitting and Parameter Estimation

The UNAIDS Kenyan data for HIV/AIDS prevalence was used to fit the AGYW and ABYM model prevalence for both the sex-structured formulation described in section 2.2 and the single-sex formulation given in section 2.5.1. We considered the gender-wise annual HIV prevalence data for the years 1990 to 2018. Table 3 gives the UNAIDS HIV prevalence data summary for the AGYW and ABYM populations respectively [41].

Table 3. 1990-2001 AGYW and ABYM UNAIDS-Kenya's Prevalence Data [41]

Year	1990	1991	1992	1993	1994	1995	1996	1997	1998	1999	2000	2001
AGYW Prevalence	6.0	7.6	9.0	10.0	10.6	10.7	10.3	9.6	8.8	7.9	6.9	6.1
ABYM Prevalence	3.3	3.8	4.3	4.5	4.5	4.3	3.9	3.5	3.0	2.6	2.3	2.0

Table 4. 2002-2013 AGYW and ABYM UNAIDS-Kenya's Prevalence Data [41]

Year	2002	2003	2004	2005	2006	2007	2008	2009	2010	2011	2012	2013
AGYW Prevalence	5.4	4.8	4.3	4.0	3.7	3.5	3.4	3.3	3.2	3.1	3.0	3.0
ABYM Prevalence	1.7	1.6	1.4	1.4	1.4	1.4	1.4	1.5	1.5	1.5	1.6	1.6

Table 5. 2014-2018 AGYW and ABYM UNAIDS-Kenya's Prevalence Data [41]

Year	2014	2015	2016	2017	2018
AGYW Prevalence	2.9	2.8	2.7	2.6	2.5
ABYM Prevalence	1.6	1.6	1.6	1.6	1.5

We define the AGYW and ABYM model prevalence as follows:

$$\text{AGYW Model Prevalence} = \frac{\text{Total number of infected AGYW}}{\text{Total AGYW population}} = \frac{I_{fu} + I_{fa} + T_{fu}}{N_f}, \tag{10}$$

$$\text{ABYM Model Prevalence} = \frac{\text{Total number of infected ABYM}}{\text{Total ABYM population}} = \frac{I_{mu} + I_{ma} + T_{mu}}{N_m}. \tag{11}$$

The AGYW and ABYM model prevalence described in equations 10 and 11 are fitted to the UNAIDS HIV prevalence data given in table 3 to estimate Λ_{fu}, Λ_{fa}, μ_f, δ_f, $\tilde{\gamma}_f$, Λ_{mu}, Λ_{ma}, μ_m, δ_m, $\tilde{\gamma}_m$, ρ_{ht}, ρ_c and ρ_t parameters. Using MATLAB built in functions 'ODE45' and 'fminsearch' we estimated the listed parameters by minimizing the sum of square difference of the AGYW and ABYM model prevalence solution and the HIV prevalence data for the AGYW and ABYM populations given in equations 12 and 13 as

$$SS^f = \sum_{k=1}^{29} \left(\frac{\left[\dfrac{I_{fu}^k + I_{fa}^k + T_{fu}^k}{S_{fu}^k + S_{fa}^k + I_{fu}^k + I_{fa}^k + T_{fu}^k + T_{fa}^k} - \tilde{Q}_1^k \right]^2}{\left[Max(\tilde{Q}_2^k, \tilde{Q}_3^k) \right]^2} \right), \tag{12}$$

$$SS^m = \sum_{k=1}^{29} \left(\frac{\left[\dfrac{I_{mu}^k + I_{ma}^k + T_{mu}^k}{S_{mu}^k + S_{ma}^k + I_{mu}^k + I_{ma}^k + T_{mu}^k + T_{ma}^k} - \tilde{Q}_4^k \right]^2}{\left[Max(\tilde{Q}_5^k, \tilde{Q}_6^k) \right]^2} \right). \tag{13}$$

The time length for the years 1990 to 2018 is given as k with $\tilde{Q}_1^k$, $\tilde{Q}_4^k$ being the yearly AGYW/ABYM UNAIDS prevalence data, $\tilde{Q}_2^k$, $\tilde{Q}_5^k$ the maximum yearly AGYW/ABYM model prevalence solutions and $\tilde{Q}_3^k$, $\tilde{Q}_6^k$ the maximum yearly AGYW/ABYM UNAIDS prevalence data. S_{fu}^k, S_{fa}^k, I_{fu}^k, I_{fa}^k, T_{fu}^k, T_{fa}^k, S_{mu}^k, S_{ma}^k, I_{mu}^k, I_{ma}^k, T_{mu}^k, T_{ma}^k are numerically computed solutions at each time k.

Attitudes affecting efficacy of HIV testing rate ρ_{ht}, condom use rate ρ_c and ART adherence rate ρ_t negatively α_{ht}^f, α_c^f, α_t^f, α_{ht}^m, α_c^m, α_t^m and positively α_{ht}^{f1}, α_c^{f1}, α_t^{f1}, α_{ht}^{m1}, α_c^{m1}, α_t^{m1} are estimated whereas the exit parameter σ is calculated as 1/24 given

that the AGYW and ABYM exit the model at the age of 24 years. The best parameters estimated by model fitting and calculated parameter are given in table 6 with $\tilde{\gamma}_f = c_f\,\gamma_f$ and $\tilde{\gamma}_m = c_m\,\gamma_m$.

Table 6. Parameter Values

Parameter	Value	Unit	Source
Λ_{mu}, Λ_{ma}	60.7685, 100.9858	$year^{-1}$	Data Estimated
μ_m	0.0101	$year^{-1}$	Data Estimated
$\tilde{\gamma}_m$	2.617	$year^{-1}$	Data Estimated
δ_m	0.0090	$year^{-1}$	Data Estimated
Λ_{fu}, Λ_{fa}	61.1842, 118.1215	$year^{-1}$	Data Estimated
μ_f	0.0004	$year^{-1}$	Data Estimated
$\tilde{\gamma}_f$	3.97580754	$year^{-1}$	Data Estimated
δ_f	0.0285	$year^{-1}$	Data Estimated
σ	0.041667	$year^{-1}$	Calculated
ρ_{ht}	0.48	$year^{-1}$	Data Estimated
ρ_c	0.3	$year^{-1}$	Data Estimated
ρ_t	0.1	$year^{-1}$	Data Estimated

We used the 2012 KAIS data described in section 2.1.1 to estimate the initial population for the state variables $S_{fu}(0) = 636$, $S_{fa}(0) = 1006$, $T_{fa}(0) = 5$, $S_{mu}(0) = 694$, $S_{ma}(0) = 867$ and $T_{ma}(0) = 3$. We estimated the initial infected population for our model as $I_{fu}(0) = 54$, $I_{fa}(0) = 76$, $T_{fu}(0) = 10$, $I_{mu}(0) = 13$, $I_{ma}(0) = 26$ and $T_{mu}(0) = 5$.

In mathematical modeling, HIV/AIDS prevalence is expected to decline in the absence of controls where HIV/AIDS epidemic is established. In the absence of controls, the Kenyan youth model prevalence trends seem to steadily increase with time (see figures 8(a), 8(b)). Interestingly, the ABYM model prevalence exceeds the AGYW model prevalence when intervention is absent (see figures 8(a), 8(b)). The Kenyan youth model prevalence without control only fits the initial rise of the HIV/AIDS epidemic.

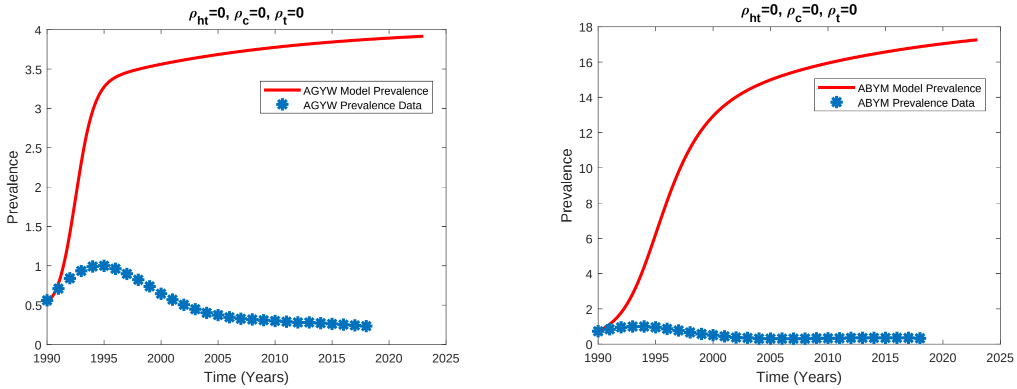

(a) AGYW model prevalence with no control (b) ABYM model prevalence with no control

Fig 8. AGYW and ABYM model prevalence with no control fitted to UNAIDS AGYW and ABYM prevalence data respectively.

While the earliest cases of HIV/AIDS in Kenya were reported in the 1980's, it was only until the late 1990's that the HIV/AIDS epidemic steadily increased from 5.3% in 1990 to a peak prevalence of 10.5% in the years 1995-1996 and by 2003, the HIV/AIDS

prevalence had declined to about 6.7% [49]. A combination of factors such as higher mortality rates, sexual behaviour change, lower incidences, delay in sexual debut among others contributed to the dramatic decline in Kenya's HIV/AIDS epidemic [49]. It is possible that even the Kenyan youth adopted safer sexual behaviors including condom use, reduction of multiple sexual partners and delay in first sex. Thus, fitting the AGYW and ABYM model prevalence to the Kenyan youth UNAIDS HIV/AIDS data subject to the estimated HIV/AIDS testing, condom use and ART adherence controls with disproportional AGYW/ABYM attitudes affecting the mentioned controls efficacy resulted in a good fit (see figures 9(a), 9(b)).

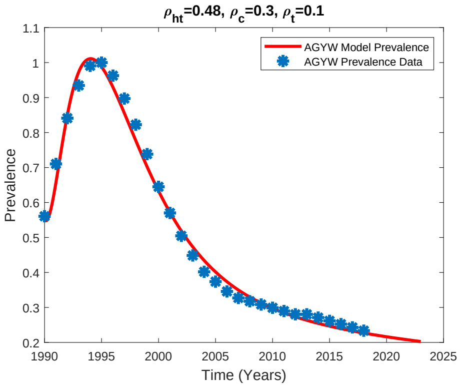

(a) AGYW model prevalence with low control

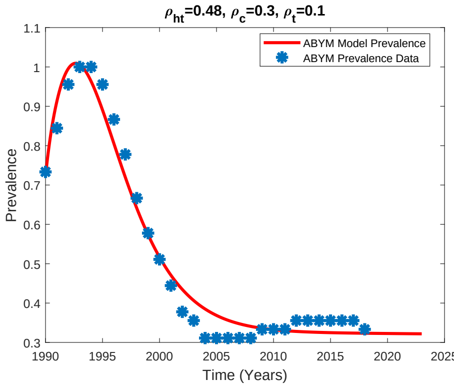

(b) ABYM model prevalence with low control

Fig 9. AGYW and ABYM model prevalence with low control fitted to UNAIDS AGYW and ABYM prevalence data respectively.

AGYW HIV/AIDS model prevalence fits well to the Kenyan UNAIDS female youth HIV/AIDS prevalence when negative attitudes towards HIV testing, condom use and ART adherence are lower in AGYW population at 18% and higher in ABYM population at 30% with positive attitudes towards the three HIV/AIDS controls greater in AGYW population at 86% compared to ABYM population which is at 69%. Similarly, ABYM model prevalence fits well when negative attitudes towards HIV/AIDS controls are greater in AGYW population at 33.7% and positive attitudes greater in ABYM population at 96%.

We used the parameter values given in table 6 to perform the numerical simulations for the model system (2) and the control reproduction number in section 2.4 with low control attitude rates given in table 7

Table 7. Estimated negative/positive attitude rates towards HIV/AIDS controls for low control simulations

Parameter	Value	Unit	Source
α^m_{ht}, α^m_c, α^m_t	0.15, 0.36, 0.38	$year^{-1}$	Estimated
α^{m1}_{ht}, α^{m1}_c, α^{m1}_t	0.99, 0.95, 0.95	$year^{-1}$	Estimated
α^f_{ht}, α^f_c, α^f_t	0.25, 0.2, 0.1	$year^{-1}$	Estimated
α^{f1}_{ht}, α^{f1}_c, α^{f1}_t	0.97, 0.8, 0.8	$year^{-1}$	Estimated

and high control attitude rates given in table 8.

Table 8. Estimated negative/positive attitude rates towards HIV/AIDS controls for high control simulations

Parameter	Value	Unit	Source
α_{ht}^{m}, α_{c}^{m}, α_{t}^{m}	0.1, 0.1, 0.1	$year^{-1}$	Estimated
α_{ht}^{m1}, α_{c}^{m1}, α_{t}^{m1}	0.9, 0.9, 0.9	$year^{-1}$	Estimated
α_{ht}^{f}, α_{c}^{f}, α_{t}^{f}	0.1, 0.1, 0.1	$year^{-1}$	Estimated
α_{ht}^{f1}, α_{c}^{f1}, α_{t}^{f1}	0.9, 0.9, 0.9	$year^{-1}$	Estimated

.

2.5.1 Single-Sex Youth Model Fit

We considered the single-sex youth model given in model system (14) to understand factors influencing its model fit. The incidence rates β_u, β_a, $\tilde{\beta}_a$ and exit rates μ_1, μ_2, ..., μ_6 are given in equation 16. See tables 9 - 10 for the single-sex model state variables and parameters description.

$$\begin{cases} \frac{dS_u}{dt} = \Lambda_u - \beta_u\, S_u - \beta_a\, S_u - \mu_1\, S_u, \\ \frac{dS_a}{dt} = \Lambda_a + \rho_{ht}\, S_u - \tilde{\beta}_a\, S_a - \mu_2\, S_a, \\ \frac{dI_u}{dt} = \beta_u\, S_u - \mu_3\, I_u, \\ \frac{dI_a}{dt} = \tilde{\beta}_a\, S_a + \beta_a\, S_u + \rho_{ht}\, I_u - \mu_4\, I_a, \\ \frac{dT_u}{dt} = \rho_{ct}\, I_a + \rho_{ct}\, T_a - \mu_5\, T_u, \\ \frac{dT_a}{dt} = \rho_{ct}^1 I_a + \rho_{ct}^1\, T_u - \mu_6\, T_a. \end{cases} \tag{14}$$

We fitted the single-sex model to the averaged AGYW/ABYM UNAIDS-Kenya HIV/AIDS prevalence data given in table 3. Using AGYW/ABYM averaged initial conditions in section 2.5 and parameter values given in table 11 yields the model fit given in figure 10(a). Adjusting the transmission risk and contact rates (see table 12) results in a good fit (see figure 10(b)).

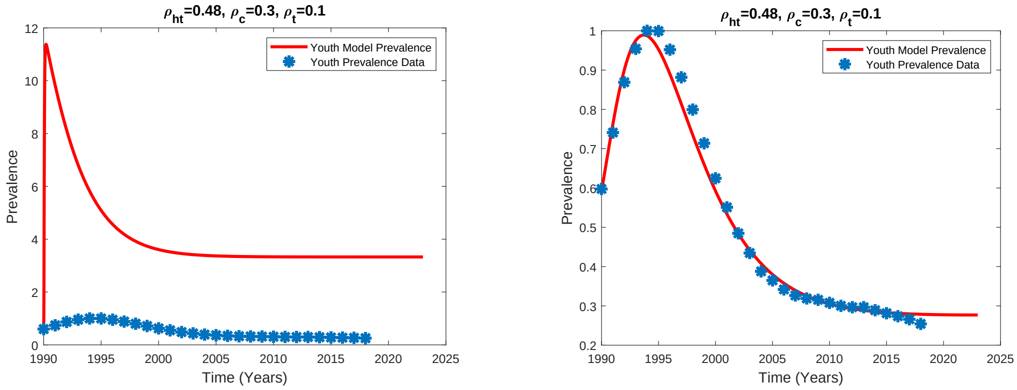

(a) Single-sex model prevalence with high transmission risk and high contact rate

(b) Single-Sex model prevalence with reduced transmission risk and reduced contact rate

Fig 10. Single-sex model prevalence with varying transmission risk and contact rate fitted to averaged UNAIDS AGYW and ABYM prevalence data.

2.6 Model Simulations

Numerical simulations on the model system equations (2) are carried out to test the AGYW and ABYM HIV/AIDS epidemic behavior. The 2020 UNAIDS 90-90-90 HIV/AIDS eradication plan aims to have at least 90% HIV/AIDS testing coverage for all persons living with HIV with at least 90% initiated on ART achieving a 90% viral load suppression [19]. This informed the 90% HIV/AIDS testing and ART efficacy rates for our high control simulations. Male condoms when used correctly and consistently in every sexual intercourse is estimated to have at least 90% efficacy against HIV/AIDS transmission whereas female condoms offer at least 94% protection [50]. Given that in the Kenyan case, male condom is most preferred as described in section 1 we used 90% condom use efficacy to model high control cases. The baseline rates for HIV/AIDS testing $\rho_{ht} = 0.48$, condom use $\rho_c = 0.3$ and ART adherence $\rho_t = 0.1$ were estimated by model fitting as described in section 2.5. Estimated constant negative/positive attitudes towards HIV/AIDS controls for the low control and high control simulations are given in tables 7 and 8 respectively.

Figures 11(a), 12(a), 13(a), 14(a) show that with time the Kenyan youth HIV/AIDS epidemic matures and attains stability without any intervention. However, the prevalence doesn't decline after attaining stability in the absence of HIV/AIDS controls (see figure 14(a)). Low control use ($\rho_{ht} = 0.48$, $\rho_c = 0.3$, $\rho_t = 0.1$) with estimated controls attitudes given in table 7 works well to reduce the infected populations and the AGYW/ABYM model prevalence with better benefits in the ABYM population (see figures 12(b), 13(b), 14(b)).

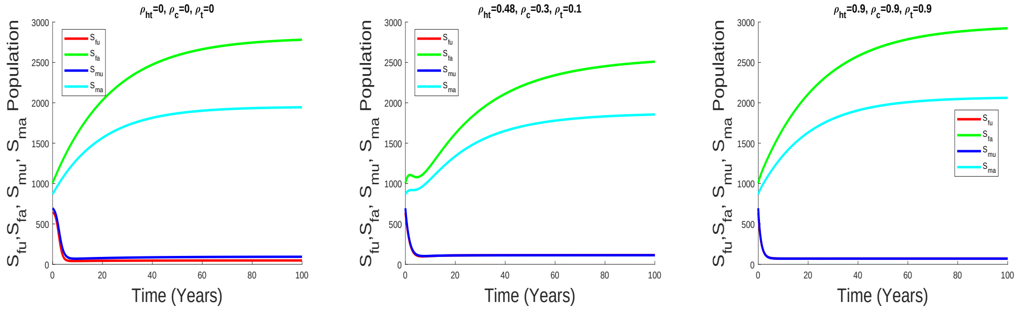

(a) (b) (c)

Fig 11. Transmission Dynamics of S_{fu}, S_{fa}, S_{mu} and S_{ma} populations with varying control .

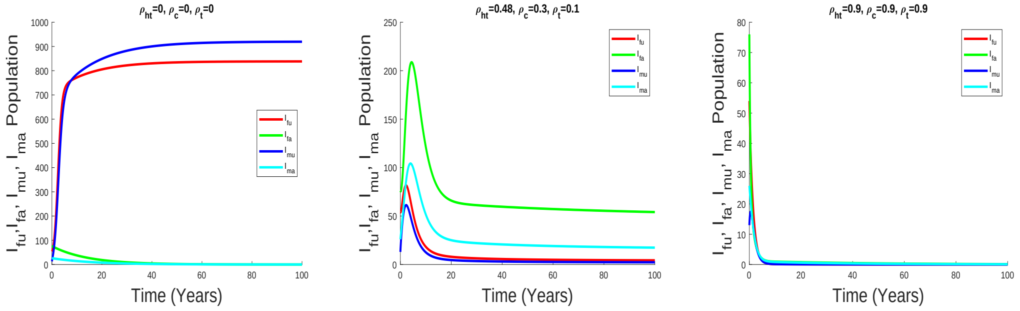

(a) (b) (c)

Fig 12. Transmission Dynamics of I_{fu}, I_{fa}, I_{mu} and I_{ma} population with varying control .

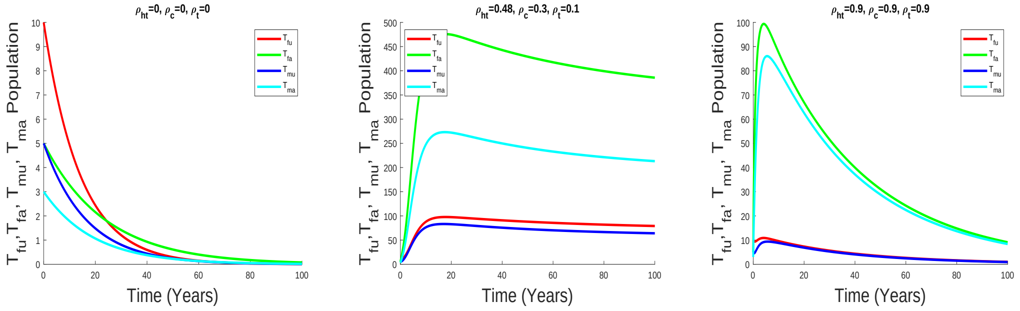

(a) (b) (c)

Fig 13. Transmission Dynamics of T_{fu}, T_{fa}, T_{mu} and T_{ma} population with varying control .

High control rates, $\rho_{ht} = 0.9$, $\rho_c = 0.9$, $\rho_t = 0.9$, with reduced negative control attitudes and increased positive control attitudes in all populations has a significant effect in HIV/AIDS disease decline among the AGYW and ABYM populations as the infected populations are reduced significantly with similar trends observed in the youth prevalence (see figures 12(c), 13(c), 14(c)). Interestingly, when the negative attitudes towards condom use and ART adherence among the AGYW and ABYM population are slightly increased when HIV/AIDS controls are low, the youth HIV/AIDS model prevalence begins to increase despite the initial decline (see figure 14(d)).

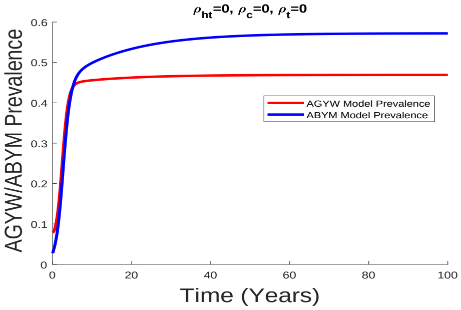

(a) No control

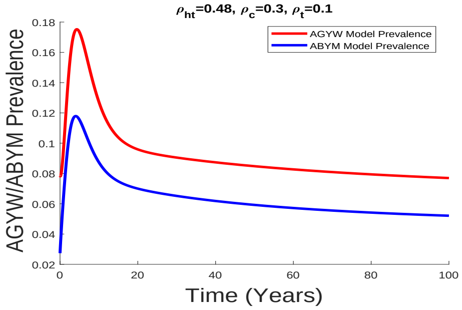

(b) Low control paired with table 7 attitudes

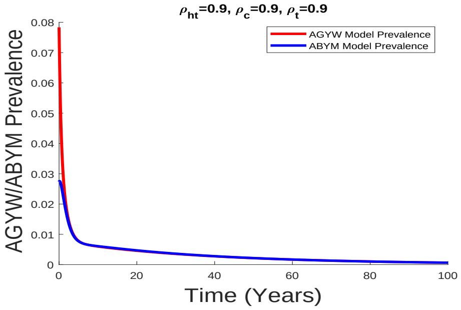

(c) High control paired with table 8 attitudes

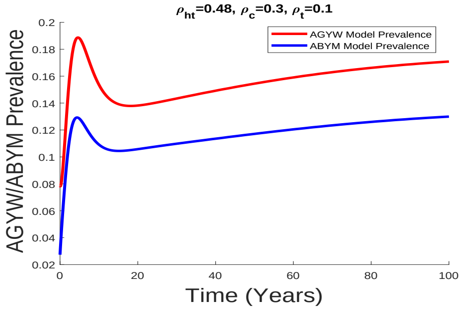

(d) Low control with increased negative attitudes towards condom use and ART adherence

Fig 14. AGYW and ABYM model prevalence with varying control.

3 Results

We investigated the effects of varying HIV/AIDS testing rates, condom use rates and ART adherence rates among the adolescent girls and young women (AGYW) and, adolescent boys and young men (ABYM) populations aged 15-24. We considered constant negative and positive attitudes influencing the uptake of HIV/AIDS controls in these populations. HIV/AIDS testing rates, condom use rates and ART adherence rates were varied from their estimated low baseline rates of 0.48, 0.3, 0.1 respectively to the estimated efficacy rates of 0.9 each. Low control simulations were associated with increased constant negative attitudes towards HIV/AIDS controls whereas high control simulations were associated with reduced negative attitudes towards HIV/AIDS controls and increased constant positive attitudes towards HIV/AIDS controls among the AGYW/ABYM populations and the Kenyan society/cultural groups. The susceptible and infected AGYW/ABYM populations were each differentiated into two broad categories according to their HIV/AIDS status knowledge. That is, uninfected aware or uninfected unaware and infected aware or infected unaware. Infected aware populations were further differentiated into two categories based on their condom use and ART adherence. Unaware populations could change their status and move to aware populations through HIV/AIDS testing, condom use or ART initiation. This model structure was largely informed by the 2012 Kenya AIDS Indicator Survey (KAIS) [40].

We fitted both the single-sex model and the sex-structured model to UNAIDS-Kenya HIV prevalence data for the young males and young females aged 15-24. The sex-structured HIV/AIDS model prevalence fitted well to each of their estimated UNAIDS-Kenya HIV/AIDS prevalence data when negative/positive attitudes towards HIV/AIDS controls were disproportional in the AGYW/ABYM populations whereas the single-sex

model prevalence trend was sensitive to transmission risk and contact rate. The single sex-structured model suggests that reduced transmission risk and sexual contact rate in the presence of low control could have resulted in reduced HIV/AIDS prevalence among the youth in Kenya. The sex-structured model further revealed the effects of disproportional gender-wise attitudes towards HIV/AIDS controls affecting uptake of controls in the AGYW/ABYM populations. Increased ABYM infectivity and reduced AGYW infectivity resulted in the female youth model good fit whereas increased AGYW infectivity and reduced ABYM infectivity resulted in the male youth good model fit. In addition to reduced transmission risk and contact rate, it is clear that gender-wise attitudes towards HIV/AIDS controls played a role in reducing HIV/AIDS prevalence among the youth in Kenya. The AGYW/ABYM model fit estimated the best parameters for model simulations.

Simulations on the control reproduction number revealed the impact of reduced transmission potential of the control reproduction number but not below unity when HIV/AIDS testing rate was fixed at a high efficacy rate of 0.9 with increasing condom use and ART adherence to high efficacy rates. This was as a result of the complex sexual structure among the Kenyan youth with the HIV/AIDS disease being sustained at endemic levels by the unaware youth. Significant HIV/AIDS reduction among the Kenyan youth will only be possible if for each sexual relationship established, there is at least one partner who is willing to disclose his/her HIV/AIDS status to his/her sex partner as well as use protection consistently. Numerical simulations on our model system revealed the impact of successful combinatory control approach in drastically reducing new HIV/AIDS infection. Low combinatory control approach has a positive effect in reducing youth disease prevalence with better benefits in the ABYM population provided the negative attitudes towards HIV/AIDS control are kept in check. Slight increase in negative attitudes towards AGYW/ABYM condom use or ART adherence can easily increase the youth disease prevalence even after the initial disease decline. Significant HIV/AIDS disease reduction is achieved only when positive attitudes towards HIV/AIDS controls are increased in all AGYW/ABYM populations with decreasing negative attitudes.

4 Discussion

Globally, male and female youth are central in the HIV/AIDS action plans due to the high numbers of youth unaware of their HIV/AIDS status [2, 51]. The 2012 Kenya AIDS Indicator Survey (KAIS) also revealed a worrying trend of many infected male and female youth unaware of their HIV/AIDS status and this is consistent with the global trends [40, 51]. The social attitudes influencing HIV/AIDS testing, condom use and ART adherence efficacy cannot be downplayed as they play a critical role in either fueling the HIV/AIDS epidemic or curtailing its spread in this population group as evidenced by the model results. The female youth HIV/AIDS prevalence trend is directly linked to increased male infectivity with decreased female infectivity while the male youth prevalence trend is directly associated with increased female infectivity and reduced male infectivity.

Annual increase of new HIV/AIDS infection in this population group exceeds HIV/AIDS related deaths which in turn increases the net size of HIV/AIDS infected population in the country [52]. This remains a huge concern as the HIV/AIDS infected youth population continues to increase, the risk of HIV/AIDS transmission increases too. Kenya's HIV/AIDS response is quite dynamic and there is increased efforts in scaling up HIV/AIDS testing, condom use and ART adherence among the AGYW and ABYM

populations. The model results reflect the importance of addressing the social attitudes inhibiting efficacy of HIV/AIDS testing, condom use and ART adherence among the Kenyan youth. While combinatory control plays a huge role in reducing HIV/AIDS prevalence trends among the youth in Kenya, the disease may still remain endemic provided the infected unaware populations sexual interactions exist. It is thus necessary to scale up HIV/AIDS testing among the youth while at the same time addressing factors affecting its efficacy such as perceived individual's risk to HIV/AIDS infection, HIV/AIDS knowledge, education, inadequate health services among others. It is also necessary to address the societal norms, psycho-social conditions, stigma, socio-cultural factors associated with condom use and ART adherence among the young people in Kenya. Their negative influence is possibly responsible for reversing decades of successful control efforts geared at reducing HIV/AIDS prevalence in Kenya.

As far as we know, there are no existing mathematical models that have addressed the impact of combinatory control and its influences among the adolescents and young adults HIV/AIDS disease dynamics in Kenya with differentiated HIV/AIDS status knowledge. Multiple control strategies such as HIV/AIDS screening, ARV drug treatment and condom use in a homogeneous population was considered by [34] to understand the potential impact on the current HIV/AIDS controls. Their results reflected the projections of HIV/AIDS epidemic trends when controls and multiple sex partners varied. Study by [34] was equivalent to the single-sex structured we considered which further revealed the effects of transmission risk and contact rate in informing the Kenyan youth HIV/AIDS prevalence trends. Considering the controls could not fit the sex-structured model prevalence, we discovered that the gender-wise effects of the social attitudes towards HIV/AIDS controls further informed the prevalence trends in the Kenyan youth HIV/AIDS dynamics.

Having studied the impact of combinatory control strategies and constant negative/positive attitudes influencing the controls efficacy among the AGYW/ABYM infected populations in a single patch model, it will be interesting to study the combinatory control effects in a metapopulation model in Kenya given that this population group is highly mobile. Dynamic attitudes towards HIV/AIDS controls should also be considered. While this study focused on population dynamics of the AGYW/ABYM, it will be interesting to study the individual based model for this AGYW/ABYM formulation. Given the behavior heterogeneity among the AGYW/ABYM, studying each individual behavior explicitly to population level could give deeper insights in understanding the social drivers of HIV/AIDS disease among the Kenyan youth. This in turn will help influence relevant policies geared at eradicating new HIV/AIDS infection among the adolescent and young adult populations in Kenya

5 Supporting information

S1 Appendix. Endemic Equilibrium Expressions

Expressions for $g_{00}, g_{01}, ..., g_{11}$, $q_{01}, q_{02}, ..., q_{20}$, $h_{01}, h_{02}, ..., h_{20}$, $C_1, C_2, ..., C_5$ and $C_{11}, C_{21}, ..., C_{51}$ in section 2.3.3.

$$
\begin{cases}
g_{00}=\dfrac{\rho_{ct}^{f1}\,\mu_{f5}+\rho_{ct}^{f1}\,\rho_{ct}^{f}}{\mu_{f5}\mu_{f6}-\rho_{ct}^{f1}\,\rho_{ct}^{f}},\ \ g_{01}=\dfrac{\rho_{ct}^{f}(1+g_{00})}{\mu_{f5}},\ \ g_{02}=1+\rho_{ht}^{m},\ \ g_{03}=\dfrac{\Lambda_{fa}\,\rho_{ht}^{m1}}{\mu_{f4}},\ \ g_{04}=\dfrac{\Lambda_{fu}\,\rho_{ht}^{m1}\rho_{ht}^{f}}{\mu_{f4}},\\
g_{05}=\dfrac{\Lambda_{fu}\,\rho_{ht}^{m}}{\mu_{f4}}+\dfrac{\Lambda_{fu}\,\rho_{ht}^{f}}{\mu_{f3}\,\mu_{f4}},\\
g_{06}=\dfrac{\rho_{ct}^{m1}\,\mu_{m5}+\rho_{ct}^{m1}\,\rho_{ct}^{m}}{\mu_{m5}\mu_{m6}-\rho_{ct}^{m1}\,\rho_{ct}^{m}},\ \ g_{07}=\dfrac{\rho_{ct}^{m}(1+g_{06})}{\mu_{m5}},\ \ g_{08}=1+\rho_{ht}^{f},\ \ g_{09}=\dfrac{\Lambda_{ma}\,\rho_{ht}^{f1}}{\mu_{m4}},\ \ g_{10}=\dfrac{\Lambda_{mu}\,\rho_{ht}^{f1}\rho_{ht}^{m}}{\mu_{m4}},\\
g_{11}=\dfrac{\Lambda_{mu}\,\rho_{ht}^{f}}{\mu_{m4}}+\dfrac{\Lambda_{mu}\,\rho_{ht}^{m}}{\mu_{m3}\,\mu_{m4}},\\
q_{01}=N_f^{*}(\bar{\mu}_f+\delta_f)-(\Lambda_{fu}+\Lambda_{fa}),\ \ q_{02}=q_{01}\,g_{02}\,\rho_{ht}^{f},\\
q_{03}=(g_{02}\,q_{01}\,\mu_{f2}+q_{01}\,\mu_{f1}\rho_{ht}^{f})-(\Lambda_{fu}\,\rho_{ht}^{f1}\,\delta_f+\Lambda_{fa}\,g_{02}\,\delta_f),\\
q_{04}=q_{01}\,\mu_{f1}\,\mu_{f2}-(\Lambda_{fu}\,\mu_{f2}\,\delta_f+\Lambda_{fa}\,\mu_{f1}\,\delta_f+\rho_{ht}^{f}\,\Lambda_{fu}\,\delta_f),\ \ q_{05}=\delta_f\,\rho_{ht}^{f}\,g_{00}\,g_{02},\\
q_{06}=g_{00}\,g_{02}\,\delta_f\,\mu_{f2}+g_{00}\,\delta_f\,\mu_{f1}\,\rho_{ht}^{f},\ \ q_{07}=g_{00}\,\delta_f\,\mu_{f1}\,\mu_{f2},\\
q_{08}=g_{02}\,g_{03}\,\rho_{ht}^{m1}+g_{05}\,\rho_{ht}^{m}\,\rho_{ht}^{m1},\\
q_{09}=g_{02}\,g_{03}\,\mu_{f2}+g_{03}\,\mu_{f1}\,\rho_{ht}^{m1}+g_{05}\,\mu_{f1}\,\rho_{ht}^{m1}+g_{05}\,\mu_{f2}\,\rho_{ht}^{m}+g_{04}\,\rho_{ht}^{m},\\
q_{10}=g_{03}\,\mu_{f1}\,\mu_{f2}+g_{05}\,\mu_{f1}\,\mu_{f2}+g_{04}\,\mu_{f2},\ \ q_{11}=g_{02}\,\rho_{ht}^{m}\,\rho_{ht}^{m1},\\
q_{12}=g_{02}\,\mu_{f2}\,\rho_{ht}^{m}+g_{02}\,\mu_{f2}\,\rho_{ht}^{m1}+\mu_{f1}\,\rho_{ht}^{m}\,\rho_{ht}^{m1},\\
q_{13}=g_{02}\,\mu_{f2}^{2}+\mu_{f1}\,\mu_{f2}\rho_{ht}^{m}+\mu_{f1}\,\mu_{f2}\rho_{ht}^{m1},\ \ q_{14}=\mu_{f1}\,\mu_{f2}^{2},\\
q_{15}=q_{08}\,q_{05}-q_{02}\,q_{11},\ \ q_{16}=q_{06}\,q_{08}+q_{05}\,q_{09}-(q_{02}\,q_{12}+q_{03}\,q_{11}),\\
q_{17}=q_{05}\,q_{10}+q_{06}\,q_{09}+q_{07}\,q_{08}-(q_{02}\,q_{13}+q_{03}\,q_{12}+q_{04}\,q_{11}),\\
q_{18}=q_{06}\,q_{10}+q_{07}\,q_{09}-(q_{02}\,q_{14}+q_{03}\,q_{13}+q_{04}\,q_{12}),\\
q_{19}=q_{07}\,q_{10}-(q_{03}\,q_{14}+q_{04}\,q_{13}),\ \ q_{20}=q_{04}\,q_{14},\\
h_{01}=N_m^{*}(\bar{\mu}_m+\delta_m)-(\Lambda_{mu}+\Lambda_{ma}),\ \ h_{02}=h_{01}\,g_{08}\,\rho_{ht}^{m},\\
h_{03}=(g_{08}\,h_{01}\,\mu_{m2}+h_{01}\,\mu_{m1}\rho_{ht}^{m})-(\Lambda_{mu}\,\rho_{ht}^{m1}\,\delta_m+\Lambda_{ma}\,g_{08}\,\delta_m),\\
h_{04}=h_{01}\,\mu_{m1}\,\mu_{m2}-(\Lambda_{mu}\,\mu_{m2}\,\delta_m+\Lambda_{ma}\,\mu_{m1}\,\delta_m+\rho_{ht}^{m}\,\Lambda_{mu}\,\delta_m),\ \ h_{05}=\delta_m\,\rho_{ht}^{m}\,g_{06}\,g_{08},\\
h_{06}=g_{06}\,g_{08}\,\delta_m\,\mu_{m2}+g_{06}\,\delta_m\,\mu_{m1}\,\rho_{ht}^{m},\ \ h_{07}=g_{06}\,\delta_m\,\mu_{m1}\,\mu_{m2},\\
h_{08}=g_{08}\,g_{09}\,\rho_{ht}^{f1}+g_{11}\,\rho_{ht}^{f}\,\rho_{ht}^{f1},\\
h_{09}=g_{08}\,g_{09}\,\mu_{m2}+g_{09}\,\mu_{m1}\,\rho_{ht}^{f1}+g_{11}\,\mu_{m1}\,\rho_{ht}^{f1}+g_{11}\,\mu_{m2}\,\rho_{ht}^{f}+g_{10}\,\rho_{ht}^{f},\\
h_{10}=g_{09}\,\mu_{m1}\,\mu_{m2}+g_{11}\,\mu_{m1}\,\mu_{m2}+g_{10}\,\mu_{m2},\ \ h_{11}=g_{08}\,\rho_{ht}^{f}\,\rho_{ht}^{f1},\\
h_{12}=g_{08}\,\mu_{m2}\,\rho_{ht}^{f}+g_{08}\,\mu_{m2}\,\rho_{ht}^{f1}+\mu_{m1}\,\rho_{ht}^{f}\,\rho_{ht}^{f1},\\
h_{13}=g_{08}\,\mu_{m2}^{2}+\mu_{m1}\,\mu_{m2}\rho_{ht}^{f}+\mu_{m1}\,\mu_{m2}\rho_{ht}^{f1},\ \ h_{14}=\mu_{m1}\,\mu_{m2}^{2},\\
h_{15}=h_{08}\,h_{05}-h_{02}\,h_{11},\ \ h_{16}=h_{06}\,h_{08}+h_{05}\,h_{09}-(h_{02}\,h_{12}+h_{03}\,h_{11}),\\
h_{17}=h_{05}\,h_{10}+h_{06}\,h_{09}+h_{07}\,h_{08}-(h_{02}\,h_{13}+h_{03}\,h_{12}+h_{04}\,h_{11}),\\
h_{18}=h_{06}\,h_{10}+h_{07}\,h_{09}-(h_{02}\,h_{14}+h_{03}\,h_{13}+h_{04}\,h_{12}),\\
h_{19}=h_{07}\,h_{10}-(h_{03}\,h_{14}+h_{04}\,h_{13}),\ \ h_{20}=h_{04}\,h_{14},\\
C_1=\dfrac{q_{16}}{q_{15}},\ \ C_2=\dfrac{q_{17}}{q_{15}},\ \ C_3=\dfrac{q_{18}}{q_{15}},\ \ C_4=\dfrac{q_{19}}{q_{15}},\ \ C_5=\dfrac{q_{20}}{q_{15}},\\
\\
C_{11}=\dfrac{h_{16}}{h_{15}},\ \ C_{21}=\dfrac{h_{17}}{h_{15}},\ \ C_{31}=\dfrac{h_{18}}{h_{15}},\ \ C_{41}=\dfrac{h_{19}}{h_{15}},\ \ C_{51}=\dfrac{h_{20}}{h_{15}}.
\end{cases}
\tag{15}
$$

S2 Appendix. Single-Sex Model Description and Parameter Values

Equation 16 gives the single-sex model incidence rates and exit rates presented in equation 14.

$$
\begin{cases}
\beta_u = \dfrac{c\,\gamma}{N_y}\left[I_u + \alpha_c\rho_c I_a + (\alpha_c\rho_c + \alpha_t\rho_t)T_u\right], \\
\beta_a = \dfrac{c\,\gamma}{N_y}\left[I_u + \alpha_c\rho_c I_a + (\alpha_c\rho_c + \alpha_t\rho_t)T_u\right]\alpha_{ht}\,\rho_{ht}, \\
\tilde{\beta}_a = \dfrac{c\,\gamma}{N_y}\left[I_u + \alpha_c\rho_c I_a + (\alpha_c\rho_c + \alpha_t\rho_t)T_u\right]\alpha^1_{ht}\,\rho_{ht}, \\
\mu_1 = \rho_{ht} + \mu + \sigma,\ \mu_2 = \mu + \sigma,\ \mu_3 = \rho_{ht} + \mu + \sigma + \delta,\ \mu_4 = \rho_{ct} + \rho^1_{ct} + \mu + \sigma + \delta, \\
\mu_5 = \rho^1_{ct} + \bar{\mu} + \delta,\ \mu_6 = \rho_{ct} + \mu + \sigma.
\end{cases}
\tag{16}
$$

Table 9. Description of Single-Sex Model State variables

Variable	Description
S_u	Susceptible youth who have never tested for HIV/AIDS
S_a	Susceptible youth who have ever tested for HIV/AIDS
I_u	Infected youth who have never tested for HIV/AIDS
I_a	Infected youth who have ever tested for HIV/AIDS
T_u	Infected aware youth who are not adherent to ART or consistent condom use
T_a	Infected aware youth who are adherent to ART and use condoms consistently

S1 Table.

Table 10. Description of Single-Sex Model Parameters

Parameter	Description
Λ_u	Natural birth and maturity rate of susceptible youth unaware of their HIV status
Λ_a	Natural birth and maturity rate of susceptible youth aware of their HIV status
ρ_{ht}	Youth HIV/AIDS testing rates
ρ_t	Youth adherence rate to anti-retroviral therapy treatment
ρ_c	Youth condom use rate
μ	Natural death rate of youth respectively
γ	Probability of youth transmission risk
δ	Disease induced deaths in youth
c_m	Youth sexual contact rate
α_{ht}, α^1_{ht}	Factors negatively and positively influencing HIV/AIDS testing rate among the youth
α_c, α^1_c	Factors negatively and positively influencing condom use rate among the youth
α_t, α^1_t	Factors negatively and positively influencing ART adherence rate among the youth
σ	Exit rate of youth upon turning 24 years

S2 Table.

Table 11. Parameter Values for the Single-Sex Model, $\tilde{\gamma} = c\gamma$

Parameter	Value	Unit	Source
Λ_u, Λ_a	60.476325, 100.55365	$year^{-1}$	Data Estimated
μ	0.0095859	$year^{-1}$	Data Estimated
$\tilde{\gamma}$	3.17245525	$year^{-1}$	Data Estimated
δ	0.0095	$year^{-1}$	Data Estimated
σ	0.041667	$year^{-1}$	Calculated
ρ_{ht}	0.48	$year^{-1}$	Data Estimated
ρ_c	0.3	$year^{-1}$	Data Estimated
ρ_t	0.1	$year^{-1}$	Data Estimated
α_{ht}, α_c, α_t	0.4, 0.27, 0.1	$year^{-1}$	Estimated
α_{ht}^1, α_c^1, α_t^1	0.78, 0.8, 0.75	$year^{-1}$	Estimated

S3 Table.

Table 12. Adjusted Parameter Values for the Single-Sex Model

Parameter	Value	Unit	Source
Λ_u, Λ_a	60.476325, 100.55365	$year^{-1}$	Data Estimated
μ	0.0095859	$year^{-1}$	Data Estimated
$\tilde{\gamma}$	0.03022869	$year^{-1}$	Data Estimated
δ	0.0095	$year^{-1}$	Data Estimated
σ	0.041667	$year^{-1}$	Calculated
ρ_{ht}	0.48	$year^{-1}$	Data Estimated
ρ_c	0.3	$year^{-1}$	Data Estimated
ρ_t	0.1	$year^{-1}$	Data Estimated
α_{ht}, α_c, α_t	0.4, 0.27, 0.1	$year^{-1}$	Estimated
α_{ht}^1, α_c^1, α_t^1	0.78, 0.8, 0.75	$year^{-1}$	Estimated

S4 Table.

Acknowledgments

The authors thank the Organization for Women in Science for the Developing World (OWSD) for financing Ms. Ronoh's research visits to University of KwaZulu-Natal (South Africa) where part of this research was done, the Simons Foundation for meeting Ms. Ronoh's home institute (University of Nairobi, Kenya) tuition costs, Mawazo Institute whose support saw Ms. Ronoh attend conferences which improved this work greatly and Mr. Innocent B. Mboya of University of KwaZulu-Natal for his guidance in data analysis.

References

1. **UNAIDS. When women lead change happens: Women advancing the end of AIDS; 2017.** `http://www.unaids.org`.

2. NACC. Kenya's fast track plan to end HIV and AIDS among adolescents and young people; 2015. https://nacc.or.ke.

3. NACC. Kenya AIDS Progress Report; 2016. https://nacc.or.ke/.

4. NASCOP. National AIDS and STI Control Program (NASCOP), Ministry of Health : AIDS in Kenya; Edition 7; 2005.

5. NCAPD. Adolescent Reproductive Health and Development Policy Plan of Action, 2005-2015 Nairobi, Kenya; 2003. National Coordinating Agency for Population and National Development and Division of Reproductive Health (Ministry of Health, Kenya).

6. UNAIDS. Prevention Gap Report; 2016. http://www.unaids.org/sites/default/files/media_asset/2016-prevention-gap-report_en.pdf.

7. UNAIDS. Kenya lauches self-testing kits and PrEP; 2017. http://www.unaids.org/en/resources/presscentres/featurestories/2017/may/20170505_kenya.

8. Kabiru C, Beguy D, Crichton J, Zulu EM. HIV/AIDS among youth in urban informal (slum) settlements in Kenya: What are the correlates of and motivations for HIV testing? BMC Public Health. 2011;11.

9. Kimera E, Vindevogel S, Maeyer JD, Reynaert D, Engelen AM, Nuwaha F, et al. Challenges and support for quality of life of youths living with HIV/AIDS in schools and larger community in East Africa: a systematic review. BMC, Published Online. 2019;8.

10. Lypen KD, Lockwood ND, F Shalabi GWH, Ngugi E. 'When we are together I feel at home.' Types and sources of social support among youth newly diagnosed with HIV in Kenya: Implications for intervention. Africa Journal AIDS Res. 2015;14:275–284.

11. KNBS. Kenya Demographic and Health Survey; 2014. Key Indicators Report.

12. Karei EM, Obbuyi A, Omollo V. Community Norms About Youth Condom Use in Western Kenya: Is Transition Occuring? African Journal of Reproductive Health. 2012;16:241–252.

13. PMA. Performance Monitoring and Accountability 2020 Kenya. Detailed Indicator Report: Kenya 2014; 2015. http://www.pma.org/sites/default/files/PMAKE-DIR-2015.04.27_2_0.pdf.

14. Kabiru C, Orpinas P. Condom Use among Kenyan High School Students. PhD Thesis, University of Georgia; 2005.

15. Maticka-Tyndale E, Tenkorang EY. A multi-level model of condom use among male and female upper primary school students in Nyanza, Kenya. Social Science and Medicine. 2010;71:616–625.

16. MacPhail C, Campbell C. I think condoms are good but, aai, I hate those things: condom use among adolescents and young people in a Southern African township. Social Science and Medicine. 2001;52:1613.

17. Eaton L, Flisher AJ, Aaaro LE. Unsafe sexual behaviour in South African youth. Social Science and Medicine. 2003;56:149.

18. Gates. Mann Global Health, Healthier People. Stronger Global Health Organizations. Kenya Case Study;. http://aidsfree.usaid.gov/sites/default/files/mgh_condom_cs_kenya.pdf.

19. UNAIDS. 90-90-90 An ambitious treatment target to help end the AIDS epidemic; 2014. http://www.unaids.org/sites/default/files/media_asset/90-90-90_en.pdf.

20. Kabogo J, Muniu E, E Songok FW, et al. Evidence of reduced treatment adherence among HIV infected paediatric and adolescent populations in Nairobi at the onset of UNAIDS Universal Test and Treat Program. BMC Research Notes. 2018;11.

21. Gachanja G. A rapid assessment of post-disclosure experiences of urban HIV-positive and HIV-negative school-aged children in Kenya. PMC PubMed. 2015;3.

22. Case KK, Ghys PD, Gouws E, Eaton JW, Borquez A, Stover J, et al. Understanding the modes of transmission model of new HIV infection and its use in prevention planning. Bulletin of the World Health Organization. 2012;90:793–868.

23. SANAC. National Strategic Plan on HIV, STIs and TB 2012 -2016; 2011. http://www.nicd.ac.za/assets/files/Acrobat%20Document4.pdf.

24. Korenromp EL, Gobet B, Fazito E, Lara J, Bollinger L, Stover J. Impact and Cost of the HIV/AIDS National Strategic Plan for Mozambique, 2015-2019—Projections with the Spectrum/Goals Model. 2015;10.

25. ASSA. The Actuarial Society of South Africa (ASSA) AIDS and Demographic model; 2011.

26. Johnson L, Dorrington R. Thembisa version 4.1: A model for evaluating the impact of HIV/AIDS in South Africa; 2018. https://www.thembisa.org/content/filedl/Thembisa4_1report.

27. Hyman JM, Li J, Stanley EA. Modelling the impact of screening and contact tracing in reducing the spread of HIV. Mathematical Biosciences. 2003;181:17–54.

28. Moghadas MS, Gumel AB, Mcleod RG, Gordon R. Could condoms stop the AIDS epidemic? Journal of theoretical medicine. 2003;5:171–181.

29. Schmitz SH. Effects of treatment or/and vaccination on HIV transmission in homosexuals with genetic heterogeneity. Mathematical Biosciences. 2000;167:1–18.

30. Cui J, Sun Y, Zhu H. The impact of media coverage on the control of infectious diseases. J Dynam Differential Equations. 2008;20:31–53.

31. Tripathi A, Naresh R, Sharma D. Modelling the effect of screening of unaware infectives on the spread of HIV infection. Applied Mathematics and Computation. 2007;184:1053–1068.

32. Joshi H, Lenhart S, Albright K, Gipson K. Modelling the effect of information campaigns on the HIV epidemic in Uganda. Mathematical Biosciences and Engineering. 2008;5:757–770.

33. Kgosimore M, Lungu EM. The effects of vertical transmission on the spread of HIV/AIDS in the presence of treatment. Mathematical Biosciences and Engineering. 2006;3:297–312.

34. Nyabadza F, Mukandavire Z, Hove-Musekwa SD. Modelling the HIV/AIDS epidemic trends in South Africa: Insights from a simple mathematical model. Nonlinear Analysis: Real World Applications. 2011;12:2091–2104.

35. Omondi EO, Mbogo RW, Luboobi LS. Mathematical modelling of the impact of testing, treatment and control of HIV transmission in Kenya. Cogent Mathematics & Statistics. 2018;5.

36. Su Z, Dong C, Li P, Deng H, Gong Y, Zhong S, et al. A mathematical modeling study of the HIV epidemics at two rural townships in the Liangshan Prefecture of the Sichuan Province of China. Infectious Disease Modelling. 2016;1:3–10.

37. Adams BM, Banks HT, Davidian M, Rosenberg ES. Model Fitting and Prediction with HIV Treatment Interruption Data; 2005. https://projects.ncsu.edu/crsc/reports/ftp/pdf/crsc-tr05-40.pdf.

38. Williams BG. Fitting and projecting HIV epidemics: Data, structure and parsimony; 2014. https://arxiv.org/ftp/arxiv/papers/1412/1412.2788.pdf.

39. Nyabadza F. A mathematical model for combating HIV/AIDS in Southern Africa: will multiple strategies work? Journal of Biological Systems. 2006;14:357–372.

40. KNBS. National Data Archive (KeNADA);. http://statistics.knbs.or.ke.

41. UNAIDS. UNAIDS-Kenya HIV/AIDS Prevalence DATA; 2018. http://aidsinfo.unaids.org.

42. Mwangi M, Waruru A, Waruiru W, Gichangi A, Toroitich-Ruto C, Kim AA. Factors associated with unsafe sex among Kenyan youth: Results from a nationally representative population-based survey. East African Journal Applied Health Monitoring and Evaluation. 2018;2:25–37.

43. UNAIDS. Undetectable=Untransmittable. Public Health and HIV Viral Load Suppression; 2018. http://www.unaids.org/sites/default/files/media_asset/undetectable-untransmittable_en.pdf.

44. Birkhoff, Rota. Ordinary Differential Equations; 1989. Wiley Series.

45. Diekmann. Patch Dynamics: An Invitation to Structured Structured (Meta) Population Models. Lecture Notes in Biomathematics. 1993;96:162–175.

46. Driessche, Watmough. Reproduction numbers and sub-threshold endemic equilibria for compartmental models of disease transmission. Mathematical Biosciences. 2002;180:29–48.

47. Diekmann O, Heesterbeek JPA. Mathematical Epidemiology of Infectious Diseasess:Model Building, Analysis and Interpretation. Wiley Series in Mathematical and Computational Biology. 2000;1.

48. Williams B, Gouws E. R0 and the elimination of HIV in Africa: Will 90-90-90 be sufficient? arXiv;1304.

49. NACC. Kenya AIDS Response Progress Report; 2014. https://nacc.or.ke.

50. USAID. Condom Fact Sheet; 2015. http://www.usaid.gov/sites/default/files/documents/1864/condomfactsheet.pdf.

51. UNICEF. Young People and HIV/AIDS: Opportunity in Crisis;.

52. Kenya HIV Estimates; 2018. https://nacc.or.ke/wp-content/uploads/2018/11/HIV-estimates-report-Kenya-20182.pdf.
